# Sugar sweetened beverages attributable disease burden and the potential impact of policy interventions: a systematic review of epidemiological and decision models

**DOI:** 10.1186/s12889-021-11046-7

**Published:** 2021-07-27

**Authors:** Andrea Alcaraz, Andrés Pichon-Riviere, Alfredo Palacios, Ariel Bardach, Dario Javier Balan, Lucas Perelli, Federico Augustovski, Agustín Ciapponi

**Affiliations:** 1 Instituto de Efectividad Clínica y Sanitaria (IECS) /Institute for Clinical Effectiveness and Health Policy, Buenos Aires, Argentina; 2 Consejo Nacional de Investigaciones Científicas y Técnicas (CONICET), Buenos Aires, Argentina; 3 Escuela de Salud Pública, Facultad de Medicina, Universidad de Buenos Aires (UBA), Buenos Aires, Argentina

**Keywords:** Sugar sweetened beverages (SSBs), Burden of disease, Economic evaluations, Decision models, Epidemiological models, Health policies

## Abstract

**Background:**

Around 184,000 deaths per year could be attributable to sugar-sweetened beverages (SSBs) consumption worldwide. Epidemiological and decision models are important tools to estimate disease burden. The purpose of this study was to identify models to assess the burden of diseases attributable to SSBs consumption or the potential impact of health interventions.

**Methods:**

We carried out a systematic review and literature search up to August 2018. Pairs of reviewers independently selected, extracted, and assessed the quality of the included studies through an exhaustive description of each model’s features. Discrepancies were solved by consensus. The inclusion criteria were epidemiological or decision models evaluating SSBs health interventions or policies, and descriptive SSBs studies of decision models. Studies published before 2003, cost of illness studies and economic evaluations based on individual patient data were excluded.

**Results:**

We identified a total of 2766 references. Out of the 40 included studies, 45% were models specifically developed to address SSBs, 82.5% were conducted in high-income countries and 57.5% considered a health system perspective. The most common model’s outcomes were obesity/overweight (82.5%), diabetes (72.5%), cardiovascular disease (60%), mortality (52.5%), direct medical costs (57.35%), and healthy years -DALYs/QALYs- (40%) attributable to SSBs. 67.5% of the studies modelled the effect of SSBs on the outcomes either entirely through BMI or through BMI plus diabetes independently. Models were usually populated with inputs from national surveys -such us obesity prevalence, SSBs consumption-; and vital statistics (67.5%).

Only 55% reported results by gender and 40% included children; 30% presented results by income level, and 25% by selected vulnerable groups. Most of the models evaluated at least one policy intervention to reduce SSBs consumption (92.5%), taxes being the most frequent strategy (75%).

**Conclusions:**

There is a wide range of modelling approaches of different complexity and information requirements to evaluate the burden of disease attributable to SSBs. Most of them take into account the impact on obesity, diabetes and cardiovascular disease, mortality, and economic impact. Incorporating these tools to different countries could result in useful information for decision makers and the general population to promote a deeper implementation of policies to reduce SSBs consumption.

**PROSPERO protocol number:**

CRD42020121025.

**Supplementary Information:**

The online version contains supplementary material available at 10.1186/s12889-021-11046-7.

## Background

Non-communicable diseases (NCD) were responsible for more than 50% of the global health burden in 2013, accounting for 38.3 million deaths worldwide [1]. About 80% of these premature NCD deaths occur in low- and middle-income countries (LMICs) [2]. Additionally, these non-communicable diseases have a huge attributable cost on health systems as well as to the society as a whole [3–8]. This enormous disease burden represents a major barrier to the achievement of the Millennium Development Goals [2].

Obesity is an important determinant of the burden of disease currently attributable to NCDs. In 2015, nearly 110 million children and more than 600 million adults were obese [9]. Since 1980, the prevalence of obesity has doubled in more than 70 countries and has continuously increased in most other countries [10]. The proportion of overweight or obese adults increased between 1980 and 2013 from 28.8 to 36.9% in men, and from 29.8 to 38.0% in women around the world [11]. Obesity-attributable diseases cause more than 17 million global deaths each year [9, 12]. The rates of childhood overweight and obesity have increased across all age and socioeconomic status (SES) groups. These trends have been remarkable in highly urbanized areas. Obese children are at increased risk of type 2 diabetes, high blood pressure, asthma, sleep disorders, liver disease, low self-esteem, depression and social isolation, and obese adults are more prone to cardiovascular diseases (CVD) and obesity-related cancers [13, 14].

Obesity is a multi-causal phenomenon, that includes unhealthy dietary patterns and sedentarism, among others. Dietary surveys indicate that foods and beverages high in free sugars can constitute a major source of discretionary calories: added sugars supply food energy but no other nutrients (also called “empty calories”).

Sugar sweetened beverages (SSBs) consumption has been linked to an increased risk of type 2 diabetes, obesity [13–15], obesity-related cancers [16], hypertension [17], coronary heart disease [15], and tooth decay [18–23]. Dental burden can also represent 5–10% of health-care budgets in industrialized countries, and even more so in low-income countries [24, 25].

Worldwide, it has been estimated that 184,000 deaths per year could be attributable to SSBs consumption: 133000 from diabetes mellitus, 45,000 from CVD, and 6450 from cancers [26]. .SSB consumption varies considerably by geographic location, gender, age and socio-economic status. The mean daily SSB consumption among adults was estimated at 137 mL (95% CI: 88 to 211 mL) in 2010 and is usually higher younger persons, low-income groups and among males [26], with large disparities between countries. These beverages include soft drinks, sodas, fruit drinks, sweetened coffees and teas, energy drinks, sports drinks, and sweetened waters. SSB constitutes the single largest source of added sugars in the American diet, and over 5% of overall caloric intake [27, 28]. Although full-calorie beverage consumption is declining, beverage consumption as a whole is increasing, especially with the mid-calorie drinks (e.g., sports drinks, teas, and energy drinks) [29].

Although many countries around the world are considering, or have begun to implement, a series of measures aimed at tackling SSB consumption, there is also a lack of awareness of this topic from decision-makers, stakeholders and the general population. Furthermore, many interventions (such as tax increases) meet with resistance. There is a wide range of interventions wherein decision makers and key leaders of various sectors are involved; the spectrum includes fiscal policies taxings SSB, front of package regulations, educational measures, modification of the school environment, publicity bans, promotion and sponsorship, among other interventions [30–34]. .Having information on the burden of disease such as the impact on health and the economics of SSB consumption as well as cost-effectiveness and the expected impact of implementing public health policies could facilitate moving forward [22].

Assessments based on epidemiological and decision models are widely accepted as decision-making tools and can provide valuable information for optimizing the allocation of health resources [35].

This study is part of a larger multi-country study funded by the International Development Research Centre (IDRC) and oriented to empowering healthcare decision makers to achieve regional needs in SSB policies in Latin America and the Caribbean through the evaluation of disease and economic burden, as well as the cost-effectiveness of available interventions.

The purpose of the study was to identify epidemiological or decision models to assess the burden of disease attributable to SSB consumption or the cost-effectiveness of interventions aimed at reducing SSB consumption, to describe the different methodological approaches through a systematic review. In other words, which are the best available published models applicable to assess SSB related problems?

## Methods

A systematic review of the published literature was carried out according to the reporting parameters proposed in the guidelines: Preferred reporting items for systematic reviews and meta-analyses (PRISMA) [36].

We undertook a systematic search up to August 2018 in the following biomedical bibliographic databases: MEDLINE (Ovid), Cochrane (Wiley), EMBase (Elsevier), CINAHL (EBSCO), LILACS (iAH). Details about the performed electronic searches are provided in the Additional file 1. Search strategy. For further information we also hand-searched reference-lists of published systematic reviews (SR) of models and performed a prospective citation tracking.

### Selection process, eligibility, and risk of bias (quality) assessment

Pairs of reviewers independently selected articles, initially by title and abstract and subsequently by evaluating the full texts of studies meeting the inclusion criteria, using the software Covidence [37, 38]. For article eligibility, the following criteria were established: 1) epidemiological or decision models exploring SSBs-related disease burden which report attributable deaths and at least one of the following outcomes: Disability-Adjusted Life Years (DALYs), Quality-Adjusted Life Years (QALYs), and Years of Life Lost (YLLs), 2) model-based economic evaluations of health interventions or policies, implemented or implementable at the city, state, or national level, and 3) descriptive studies of decision models that explore disease burden or cost-effectiveness. The exclusion criteria were: 1) publishing date before the year 2003 -since the relationship between SSBs and health risk was not previously clear-, 2) cost-only studies (health effects not included), 3) models not specifically about SSBs or which do not distinctively show SSBs effects, 4) economic evaluations based only on randomized controlled trials (piggyback studies). Based on the paper by Brennan et al. paper [39] we defined a model as a formal quantified comparison, which summarize sources of evidence on costs and benefits, in order to identify the best option for decision makers to adopt. These authors additionally proposed a model taxonomy according to different dimensions. We simplified this taxonomy to account for different model characteristics: whether they incorporated interactions between the individuals; whether they were epidemiological (a simple usually excel based model), aggregated (nonindividual) or at individual level; how they handled the time variable (as a continuous variable, as discrete steps/cycles or were untimed); or were based on cohorts (state-transition/Markov models).

Pairs of reviewers independently extracted data using a previously piloted data extraction form and assessed the risk of bias (quality) of the included studies. In case of disagreements, it was resolved by consensus. If reaching a consensus proved to be difficult, a third author made the final decision. Considering the nature of our research question, a specific risk of bias (quality) assessment tool was not deemed to be applicable. Nevertheless, we used the items in the data extraction template to assess the exhaustiveness of the model features and description.

### Data synthesis

We performed a descriptive synthesis of the main characteristics of the identified models.

For each model we considered its type, frequency of use in public health, specificity for SSBs- attributable effects, time horizon, perspective, age, sex, SES and countries of application to allow subgroup analyses. We assessed the following features: presence of interaction between individuals, degree of information aggregation (individual, aggregate, econometric or epidemiological), temporal dimension incorporation, and number of cohorts required (single cohort vs multicohort). Regarding model inputs, we specified data requirements such as incidence, data by condition, vital data, longitudinal data, representative surveys, or other data.

As for outcomes, we analyzed if they reported: variations in the degree of consumption, obesity/overweight, diabetes, mortality, cardiovascular disease, cancer, tooth cavities, bullying, other health outcomes; DALYs, QALYs or YLL; direct or indirect costs; tax revenue, sales of sugary drinks and health equity aspects. For cost-effectiveness models we also considered which type of intervention was being evaluated: taxation, school environment modifications, advertising, labeling, or others. We estimated the workload needed to complete populating models according to their type, as well as their applicability to the Latin-American and Caribbean context. In all cases they scored as low, medium or high effort /applicability and the final decision was reached by group consensus.

Finally, we identified each model’s underlying disease causal pathway and graphically assigned it to a group according to its pathway and / or the manner in which results were reported.

## Results

**Fig. 1:**
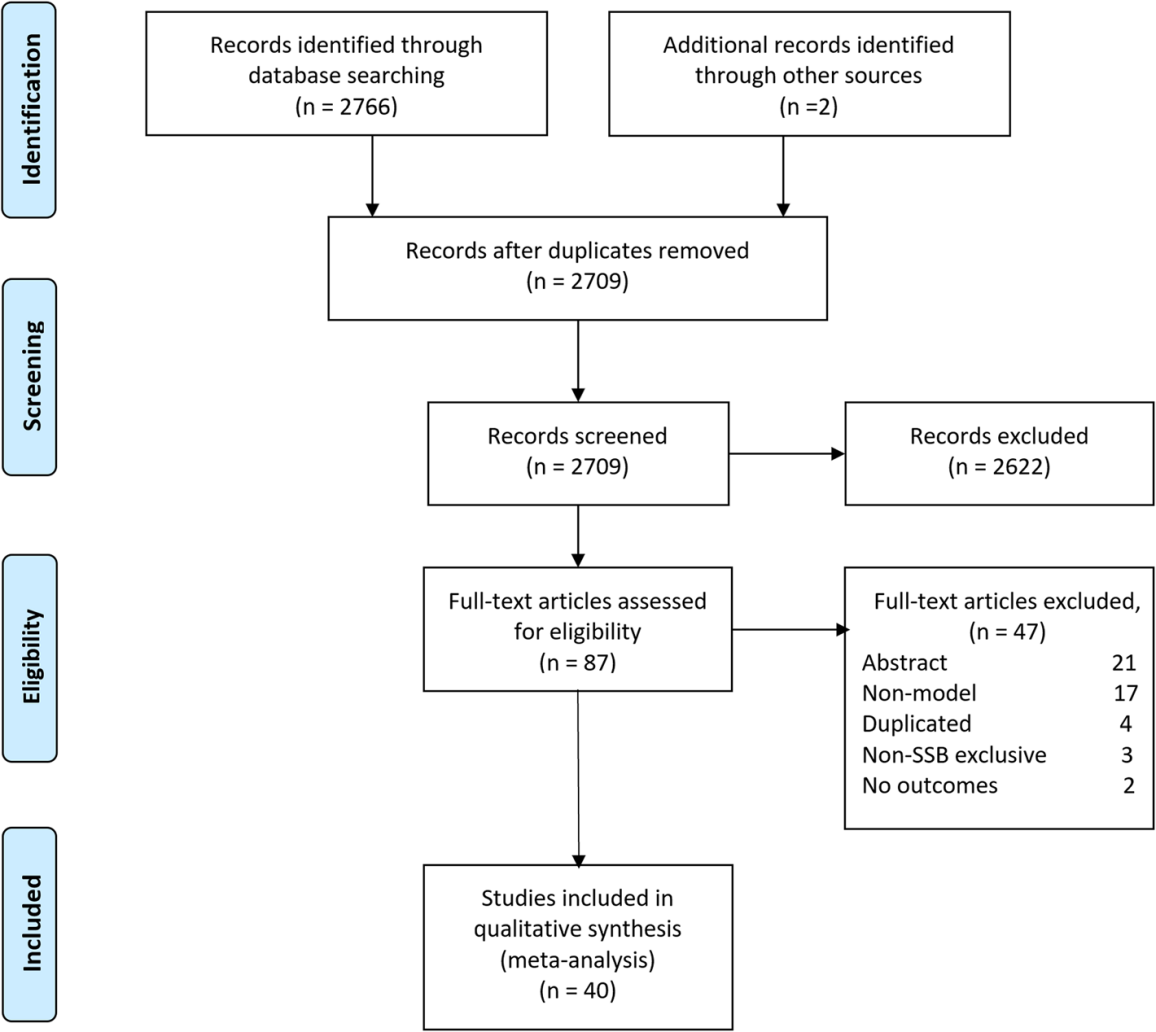
Flow diagram of studies in the systematic review. Note: Abstract refers to articles classified as not meeting the inclusion criteria through its abstract. Duplicated refers to the fact that it is exactly the same study. Non-SSBs exclusive refers to models that do not allow differentiating the exclusive effects of SSBs. No outcomes: the study don’t report the outcomes of interest.

We identified a total of 2766 references from the bibliographic databases (2709 after removal of duplicates). We selected 87 of those for eligibility by full text-screening and we finally included 40 studies [18, 23, 25–27, 32, 40–73] published between 2012 and 2018 (See Fig. 1. Study flow diagram).

The descriptive statistics of the characteristics of the included SSBs models are described in Tables 1 and 2. A detailed description of each model’s features, taxonomy, inputs, results, subgroups analyzed, interventions evaluated, and applicability can be found in Additional file 2. Tables 1 to 5. These characteristics were considered as an indirect proxy of the study quality.

**Table 1:** Descriptive statistics of the included SSBs models: features, taxonomy, and applicability

Model	Descriptive variables	Frequency (*N* = 40 studies)	%
**Features**	Specific to SSBs	*N* = 18	45.0%
	Time horizon (maximum)	1 year = 8	22.5%
		2–10 years = 14	35.0%
		11–25 years = 9	22.5%
		Lifetime = 8	20.0%
	Population	Adults only = 21	52.5%
		Childs only = 2	5.0%
		Total population = 17	42.5%
	Country by income	High income = 30	75,00%
		Low and middle income = 9	22,50%
		Worldwide = 1	2,50%
	Perspective	Government = 6	15.0%
		Health system = 23	57.5%
		Societal = 11	27.5%
**Taxonomy**	Interaction allowed	*N* = 0	0%
	Aggregate/ Individual/ econometric/ epidemiological	Aggregate = 14	35.0%
		Individual = 1	2.5%
		Econometric = 8	20.0%
		Epidemiological = 17	42.5%
	Time incorporation	Timed = 20	50.0%
		Untimed = 13	32.5%
		Continuous =6	15.0%
		Not applicable = 1	2.5%
	Cohort	Cohort =25	62.5%
		Multi-cohort = 8	20.0%
		Not reported/applicable = 7	17.5%
**Applicability**	Effort / requirements	Low = 4	10.0%
		Moderate = 34	85.0%
		High = 2	5.0%
	Applicability / reproducibility	Moderate = 35	87.5%
		High = 5	12.5%

**Table 2:** Descriptive statistics of the included SSBs models: inputs, results, subgroups, and interventions

Model	Descriptive variables	Frequency (*N =* 40 studies)	%
**Inputs**	Incidence	*N* = 21	52.5%
	Vital statistics	*N* = 27	67.5%
	Longitudinal data	*N* = 9	22.5%
	Population survey	*N* = 37	87.5%
	Demand elasticity	*N* = 11	27.5%
**Results**	Obesity/Overweight	*N* = 36	90.0%
	Diabetes	*N* = 29	72.5%
	Cardiovascular disease	*N* = 31	77.5%
	Cancer	*N* = 13	32.5%
	Cavities	*N =* 2	5.0%
	Osteoarthritis	*N =* 2	5.0%
	Incidence	*N =* 3	7.5%
	Prevalence	*N* = 6	15.0%
	Mortality	*N =* 21	52.5%
	Life years	*N =* 11	27.5%
	DALYs/QALYs	*N* = 16	40.0%
	Direct costs	*N* = 23	57.5%
	Indirect costs	*N =* 6	15.0%
	Cost-effectiveness	*N* = 7	17.5%
	Variation in consumption	*N* = 34	85.0%
	SSBs sales	*N =* 7	17.5%
	Tax collection	*N =* 7	17.5%
	Equity	*N =* 13	32.5%
**Subgroups**	Children/teenage	*N =* 16	40.0%
	Gender	*N =* 22	55.0%
	Income level	*N* = 12	30.0%
	Vulnerable groups	*N* = 10	25.0%
**Interventions evaluated**	Taxes	*N* = 30	75.0%
	School environment	*N* = 5	12.5%
	Advertising	*N =* 4	10.0%
	Labelling	*N =* 2	5.0%
	Subsidies	*N* = 2	5.0%

*SSBs* sugar sweetened beverages. Demand elasticity is an economic measure of the sensitivity of demand of SSBs relative to a change in another variable, usually the price. Vulnerable groups: ethnicity, rural status, literacy, education level or participants of a nutritional assistance program

Out of the 40 included studies only 45% were exclusive for SSBs, while in the rest of the models SSBs was one of the risk factors evaluated among others (e.g., obesity or cardiovascular risk factors). 72.5% analyzed a maximum time horizon of 10 or more years; only 25% considered child population separately. Only 12.5% included a Latin-American country while 82.5% were conducted in high income countries, mainly in USA, Australia and the UK. Most models were based on healthcare system perspective (57.5%).

Regarding the model taxonomy, none incorporated interactions between the individuals; most were classified as epidemiological (42.5%), aggregated -non individual- level (35.5%), timed (50%), and based on cohort (62.5%). A detailed description of each model can be found in Additional file 2. Table 6.

The majority of the models involve moderate applicability efforts or requirements (85%) and show moderate applicability to Latin-America and the Caribbean region (87.5%).

Most models required inputs from national surveys -such as obesity prevalence, SSBs consumption-; vital statistics (67.5%) mainly related to specific disease mortality; and each country’s disease incidence (52.5%). See Table 1.

The most common results provided by the burden of disease models were obesity/overweight (82.5%), diabetes (72.5%), cardiovascular disease (60%), mortality (52.5%), direct medical costs (57.35%), and DALYs/QALYs (40%) attributable to SSBs. Only two models incorporate diseases not related to obesity such as cavities. Equity analysis was considered in one third of the studies. Models that evaluated the impact of interventions also included the variation in SSBs consumption (85%) but only 17.5% reported tax collection or SSBs sales.

Regarding the analysis of different population subgroups, 55% reported results by gender and 40% included children; only 30% of the models presented results by income level, and only 25% by vulnerable groups.

Most of the models evaluated at least one intervention (92.5%), taxes being the most evaluated intervention (75%) followed by school environments (12.5%) and advertising (10%). See Table 2.

**Fig. 2:**
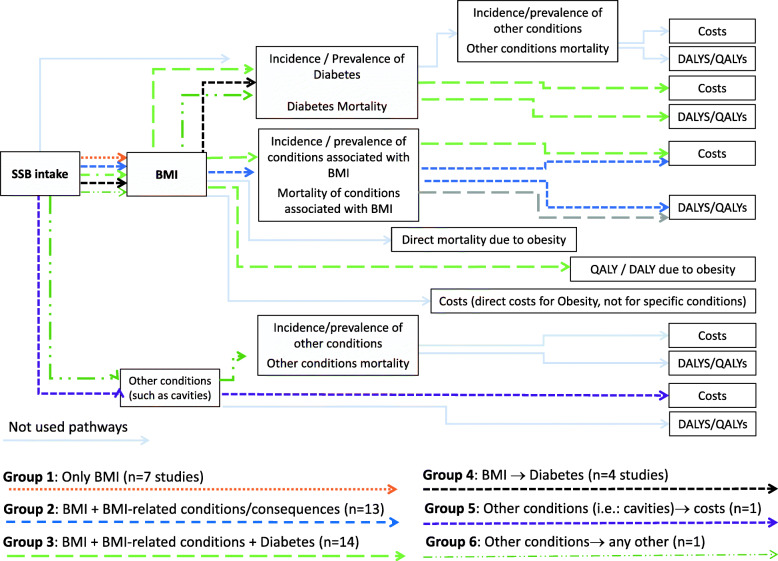
Model pathways groups

After analyzing each model causal (structural) pathway, we grouped them in six main pathway patterns, which are graphically presented in Fig. 2 Models’ pathways groups; complemented by the data in Table 3. The most frequent pathway (35%) included the effects of SSBs intake on BMI with the mortality and quality of life of the obesity plus the effects of diabetes and other related diseases/conditions including mortality, DALYS/QALYs and the cost associated with their treatment. A similar pathway that was almost as frequent (32.5%) did not consider diabetes independently, but it was included with all the other conditions (mainly cardiovascular disease and cancer) (see Fig. 2, groups 2 and 3). Models that used the simplest pathway, which only include SSBs intake to BMI represent 17.55% of the cases. Other pathways were less frequent or not used at all. Each model pathway is graphically represented in figures in Additional file 3. Pathways by study.

**Table 3:** Disease pathway pattern groups of the included SSBs models

Pathway pattern		N (%)	Studies ID	References
**Group 1**	Only BMI	7 (17.5%)	Briggs 2013a, Briggs 2013b, Kristensen 2014, Lee 2018, Manyema 2014, Vecino-Ortiz 2018, Wilson 2015.	[25, 46, 47, 55, 57, 62, 72]
**Group 2**	BMI + BMI-related conditions / consequences	13 (32.5%)	Collins 2015, Gortmaker 2015b, Gortmaker 2015a, Lin 2011, Long 2015, Manyema 2015, Manyema 2016, Nomaguchi 2017, Pearson-Stuttard 2017, Rezende 2016, Sacks 2011, Singh 2015, Wright 2015	[23, 26, 27, 32, 51, 53, 54, 59, 61, 64, 65, 67, 73]
**Group 3**	BMI + BMI-related conditions + Diabetes	14 (35.0%)	Afshin 2015, Basu 2013, Brown 2018, Breeze 2017, Briggs 2017, Cobiac 2017, Crino 2017, Lal 2017, Magnus 2016, Mekonnen 2013, Penalvo 2017, Sanchez Romero 2016, Veerman 2016, Wang 2012	[18, 40, 43, 45, 48–50, 52, 56, 61, 66, 68, 70, 71]
**Group 4**	BMI + Diabetes	4 (10.0%)	Barrientos-G. 2017, Basu 2014 a, Basu 2014b, Ma 2016	[41, 42, 44, 60]
**Group 5**	Other, such as cavities, → costs	1 (2.5%)	Schwendicke 2016	[69]
**Group 6**	Other → any other	1 (2.5%)	Lieffers 2018	[58]

## Discussion

Our systematic review offers a unique and up to date snapshot of current SSBs models, and provides a detailed description of the 40 included studies involving model features, inputs, results, pathways, interventions and applicability issues. This can significantly facilitate the use, adaptation, or development of future models that will improve the current tools aiming to implement a successful SSBs policy.

Remarkably, less than half of the models were specifically designed for SSBs, though all of them provided useful information in order to facilitate the use and adapt or develop a model in future endeavors. The information identified can be used in different contexts; we incorporated information from five continents, including some global approaches [25] and from all perspectives, such as healthcare systems, governments and society as a whole.

As to the complexity of the required input parameters, most models require available or feasible inputs, like representative population surveys -mainly for obesity prevalence-, or vital statistics for mortality by conditions; while some other inputs -such as incidence or longitudinal data- could be more difficult to obtain in many settings, though required by fewer models. This can also be true for the level of disaggregation of some parameters; for example, finding data by age by single year or by gender could be difficult to achieve in some countries or regions. Information regarding SSBs consumption could be difficult to find in Latin-American and the Caribbean countries and specific information on children is usually unavailable. Additionally, the models that evaluating the impact of a required intervention required demand elasticity for SSBs, ideally by age and gender groups, which data is not easily available in many countries.

Models offer relevant results to assess the burden of disease and / or the cost-effectiveness of interventions including the expected variation on SBBs consumption of different policies, obesity/overweight, diabetes, cardiovascular disease, and mortality. Many of the models do not report results in a sufficiently disaggregated manner, thus limiting their applicability and usefulness to end users such as decision makers. The evidence identifies presents some limitations. SSBs consumption is really dissimilar among subgroups, for example adolescents usually consumes more than adults and there are big differences between genders by ages groups or income quintiles [74]. Moreover, the prevalence of obesity and disease has different effects according to gender, age, and income [10, 75, 76]; so it is really usefully to have the opportunity to analyze the effects of SSBs in a disaggregated manner.

Direct and indirect costs and quality of life -DALYs and QALYs- are measures which usually serve as a guide for resource allocation and are valuable for decision makers; but only 57.5 and 40% of the models incorporated them, respectively. Children were usually omitted in most of the studies, even though they are a widely affected population and a high-priority target for the prevention policies advocated by international organizations such as UNICEF and by numerous health systems.

It is encouraging that most of the interventions studied are those most grounded on evidence such as taxes, school food policies and advertising [77, 78].

Our results show that a variety of specific modelling approaches to SSBs consumption has been used to understand its associated burden. Most of the published studies model the effects of SSBs consumption through increased BMI and the consequences for health -and sometimes for quality of life and the cost- implied. While sometimes the models separate the effects of diabetes, the effect through BMI is invariably considered without including the direct effect of SSBs on diabetes as well [79]. A direct effect of SSBs on cardiovascular disease has been recently recognized (independently of BMI), which no model had previously included [80]. The Australian Assessing Cost-Effectiveness (ACE) model [81] was the most frequently used model, including adaptations to the USA [50, 52–54, 56, 59, 64, 67, 70, 73]. This model is both time- and data- consuming and requires researchers and users to have a high level of understanding of modelling issues, so it is probably difficult to apply in many countries. Our review finds many other model and model causal pathways that could be used. The selection of the appropriate model for each country could depend on the availability of local data, the time horizon selected, the health policy to be evaluated, among others.

A systematic review evaluating the impact of taxes on SSBs according to socio-economic status uncovered that models are focused on SSBs consumption rather than on the burden of disease; few models evaluate the impact on BMI but most of them only evaluated the impact on SSBs consumption [82]. We similarly found out that few studies specifically disaggregated results according to income groups.

Worldwide -and more so in low- and middle-income countries- the general population and decision-makers are not yet fully aware of the dimension of the problem that an excessive SBBs consumption can cause; therefore, studies estimating the attributable disease burden are really important. Also, the interventions that need to be implemented -taxes, labeling, publicity limitation, school environment modifications- are both politically and socially sensitive, and the beverage industry frequently obstructs their implementation [83]. While SSBs taxes have been instituted in over 40 countries and cities [84], the epidemiological shift towards NCDs diseases in low- and middle-income countries (LMICs) warrants the implementation of an even stricter SSBs control policy encompassing all the effective interventions available [77, 78].

The value of non-communicable disease modelling to inform health policy is well established [85–87]. These models guide decision-makers in the implementation of policies to improve risk factors for chronic diseases. The tobacco experience has shown that the burden of disease and economic evaluations have promoted an effective WHO framework implementation all around the world [88]. Four our SSBs-related focus, we found 40 published models that attempt to assess this information on burden of disease that could guide and promote the implementation of evidence-based policies aiming to decrease SSBs consumption and its associated burden. Implementing effective SSBs policies is particularly important for LMICs with double nutritional burden of malnutrition and obesity.

Based on this information each country could select a simple or a more advanced model to apply within its boundaries and also identify what the main inputs and results that could be useful for making decisions in the local context are.

## Conclusions

There is a wide range of modelling approaches with different complexities and information requirements to evaluate the burden of disease attributable to SSBs. The majority of these approaches consider the impact on obesity, diabetes and cardiovascular disease, mortality, and economic impact. The incorporation of these tools in different countries could generate useful information for decision makers and the general population and promote a deeper implementation of policies to diminish SSBs consumption.

## Supplementary Information

**Additional file 1.** Search Strategy

**Additional file 2.** Complementary tables

**Additional file 3.** Pathways by study

## Data Availability

The datasets used and/or analysed during the current study are available from the corresponding author on reasonable request.
