# Supplementary material for: Sugar sweetened beverages attributable disease burden and the potential impact of policy interventions: a systematic review of epidemiological and decision models
Source: BMC Public Health. 2021 Jul 27;21:1460. doi: 10.1186/s12889-021-11046-7 (PMC8317409; doi:10.1186/s12889-021-11046-7)
Supplement: Supplementary file 1 — Additional file 1. Search Strategy [file 12889_2021_11046_MOESM1_ESM.docx]

**Sugar sweetened beverages attributable disease burden and the potential impact of policy interventions: a systematic review of epidemiological and decision models**

**Additional file 1. Search Strategy**

**MEDLINE** (Ovid MEDLINE(R) Epub Ahead of Print, In-Process & Other Non-Indexed Citations, Ovid MEDLINE(R) Daily and Ovid MEDLINE(R) 1946 to Present)

1 exp Models, Economic/

2 Economic Model*.ti,ab.

3 Econometric Model*.ti,ab.

4 Decision Analy*.ti,ab.

5 exp Cost-Benefit Analysis/

6 Cost Benefit.ti,ab.

7 Cost Effectiv*.ti,ab.

8 Comparative Risk.ti,ab.

9 Risk Assess*.ti,ab.

10 DALY*.ti,ab.

11 QALY*.ti,ab.

12 Policy Model.ti,ab.

13 Mathematical Model*.ti,ab.

14 Mathematical Analys*.ti,ab.

15 Public Health Model*.ti,ab.

16 Public Health Intervention*.ti,ab.

17 Modelling.ti,ab.

18 Policy Evaluation*.ti,ab.

19 exp Markov Chains/

20 Markov.ti,ab.

21 exp Monte Carlo Method/

22 Monte Carlo.ti,ab.

23 exp Regression Analysis/

24 Regression Analy*.ti,ab.

25 exp Models, Statistical/

26 Statistical Model*.ti,ab.

27 Probabilistic Model*.ti,ab.

28 or/1-27

29 Burden.ti,ab.

30 Attributable*.ti,ab.

31 Societal Cost*.ti,ab.

32 or/29-31

33 Model*.ti,ab.

34 Estimat*.ti,ab.

35 Projection*.ti,ab.

36 Projecting.ti,ab.

37 or/33-36

38 32 and 37

39 28 or 38

40 exp Carbonated Beverages/

41 Soda*.ti,ab.

42 Carbonated Beverage*.ti,ab.

43 Carbonated Drink*.ti,ab.

44 SSB.ti,ab.

45 Carbonated Drink*.ti,ab.

46 Soft Drink*.ti,ab.

47 Soft Beverage*.ti,ab.

48 Sweetened Drink*.ti,ab.

49 Sweetened Beverage*.ti,ab.

50 Sugared Drink*.ti,ab.

51 Sugary Drink*.ti,ab.

52 Sugared Beverage*.ti,ab.

53 Sugary Beverage*.ti,ab.

54 Cola.ti,ab.

55 Sport Drink*.ti,ab.

56 Sport Beverage*.ti,ab.

57 Fruit Drink*.ti,ab.

58 Fruit Beverage*.ti,ab.

59 exp "Fruit and Vegetable Juices"/

60 Juice*.ti,ab.

61 Fizzy Drink*.ti,ab.

62 Fizzy Beverage*.ti,ab.

63 or/40-62

64 exp Sweetening Agents/

65 exp Dietary Sugars/

66 Syrupe*.ti,ab.

67 Sucrose*.ti,ab.

68 Fructose*.ti,ab.

69 or/64-68

70 Beverage*.ti,ab.

71 Drink*.ti,ab.

72 70 or 71

73 69 and 72

74 63 or 73

75 exp Diabetes Mellitus/

76 Diabetes.ti,ab.

77 Diabetic*.ti,ab.

78 exp Overweight/

79 Obes*.ti,ab.

80 Overweight.ti,ab.

81 exp Body Mass Index/

82 BMI.ti,ab.

83 exp Cardiovascular Diseases/

84 Cardiovascular.ti,ab.

85 CVD.ti,ab.

86 Heart Disease*.ti,ab.

87 exp Dental Caries/

88 Dental Decay.ti,ab.

89 Caries.ti,ab.

90 exp Neoplasms/

91 Neoplas*.ti,ab.

92 Cancer.ti,ab.

93 Tumor*.ti,ab.

94 Tumour*.ti,ab.

95 Carcinom*.ti,ab.

96 Malignan*.ti,ab.

97 exp Hypertension/

98 Hypertens*.ti,ab.

99 Blood Pressure.ti,ab.

100 or/75-99

101 39 and 74 and 100

**Cochrane (Wiley)**

ID Search Hits

#1 MeSH descriptor: [Models, Economic] explode all trees

#2 Economic Model*:ti,ab,kw (Word variations have been searched)

#3 Econometric Model*:ti,ab,kw (Word variations have been searched)

#4 Decision Analy*:ti,ab,kw (Word variations have been searched)

#5 MeSH descriptor: [Cost-Benefit Analysis] explode all trees

#6 Cost Benefit:ti,ab,kw (Word variations have been searched)

#7 Cost Effectiv*:ti,ab,kw (Word variations have been searched)

#8 Comparative Risk:ti,ab,kw (Word variations have been searched)

#9 Risk Assess*:ti,ab,kw (Word variations have been searched)

#10 DALY*:ti,ab,kw (Word variations have been searched)

#11 QALY*:ti,ab,kw (Word variations have been searched)

#12 Policy Model:ti,ab,kw (Word variations have been searched)

#13 Mathematical Model*:ti,ab,kw (Word variations have been searched)

#14 Mathematical Analys*:ti,ab,kw (Word variations have been searched)

#15 Public Health Model*:ti,ab,kw (Word variations have been searched)

#16 Public Health Intervention*:ti,ab,kw (Word variations have been searched)

#17 Modelling:ti,ab,kw (Word variations have been searched)

#18 Policy Evaluation*:ti,ab,kw (Word variations have been searched)

#19 MeSH descriptor: [Markov Chains] explode all trees

#20 Markov:ti,ab,kw (Word variations have been searched)

#21 MeSH descriptor: [Monte Carlo Method] explode all trees

#22 Monte Carlo:ti,ab,kw (Word variations have been searched)

#23 MeSH descriptor: [Regression Analysis] explode all trees

#24 Regression Analy*:ti,ab,kw (Word variations have been searched)

#25 MeSH descriptor: [Models, Statistical] explode all trees

#26 Statistical Model*:ti,ab,kw (Word variations have been searched)

#27 Probabilistic Model*:ti,ab,kw (Word variations have been searched)

#28 #1 or #2 or #3 or #4 or #5 or #6 or #7 or #8 or #9 or #10 or #11 or #12 or #13 or #14 or #15 or #16 or #17 or #18 or #19 or #20 or #21 or #22 or #23 or #24 or #25 or #26 or #27

#29 Burden:ti,ab,kw (Word variations have been searched)

#30 Attributable*:ti,ab,kw (Word variations have been searched)

#31 Societal Cost*:ti,ab,kw (Word variations have been searched)

#32 #29 or #30 or #31

#33 Model*:ti,ab,kw (Word variations have been searched)

#34 Estimat*:ti,ab,kw (Word variations have been searched)

#35 Projection*:ti,ab,kw (Word variations have been searched)

#36 Projecting:ti,ab,kw (Word variations have been searched)

#37 #33 or #34 or #35 or #36

#38 #32 and #37 #39 #28 or #38

#40 MeSH descriptor: [Carbonated Beverages] explode all trees

#41 Soda*:ti,ab,kw (Word variations have been searched)

#42 Carbonated Beverage*:ti,ab,kw (Word variations have been searched)

#43 Carbonated Drink*:ti,ab,kw (Word variations have been searched)

#44 SSB:ti,ab,kw (Word variations have been searched)

#45 Carbonated Drink*:ti,ab,kw (Word variations have been searched)

#46 Soft Drink*:ti,ab,kw (Word variations have been searched)

#47 Soft Beverage*:ti,ab,kw (Word variations have been searched)

#48 Sweetened Drink*:ti,ab,kw (Word variations have been searched)

#49 Sweetened Beverage*:ti,ab,kw (Word variations have been searched)

#50 Sugared Drink*:ti,ab,kw (Word variations have been searched)

#51 Sugary Drink*:ti,ab,kw (Word variations have been searched)

#52 Sugared Beverage*:ti,ab,kw (Word variations have been searched)

#53 Sugary Beverage*:ti,ab,kw (Word variations have been searched)

#54 Cola:ti,ab,kw (Word variations have been searched)

#55 Sport Drink*:ti,ab,kw (Word variations have been searched)

#56 Sport Beverage*:ti,ab,kw (Word variations have been searched)

#57 Fruit Drink*:ti,ab,kw (Word variations have been searched)

#58 Fruit Beverage*:ti,ab,kw (Word variations have been searched)

#59 MeSH descriptor: [Fruit and Vegetable Juices] explode all trees

#60 Juice*:ti,ab,kw (Word variations have been searched)

#61 Fizzy Drink*:ti,ab,kw (Word variations have been searched)

#62 Fizzy Beverage*:ti,ab,kw (Word variations have been searched)

#63 #40 or #41 or #42 or #43 or #44 or #45 or #46 or #47 or #48 or #49 or #50 or #51 or #52 or #53 or #54 or #55 or #56 or #57 or #58 or #59 or #60 or #61 or #62

#64 MeSH descriptor: [Sweetening Agents] explode all trees

#65 MeSH descriptor: [Dietary Sugars] explode all trees

#66 Syrupe*:ti,ab,kw (Word variations have been searched)

#67 Sucrose*:ti,ab,kw (Word variations have been searched)

#68 Fructose*:ti,ab,kw (Word variations have been searched)

#69 #64 or #65 or #66 or #67 or #68

#70 Beverage*:ti,ab,kw (Word variations have been searched)

#71 Drink*:ti,ab,kw (Word variations have been searched)

#72 #70 or #71

#73 #69 and #72

#74 #63 or #73

#75 MeSH descriptor: [Diabetes Mellitus] explode all trees

#76 Diabetes:ti,ab,kw (Word variations have been searched)

#77 Diabetic*:ti,ab,kw (Word variations have been searched)

#78 MeSH descriptor: [Overweight] explode all trees

#79 Obes*:ti,ab,kw (Word variations have been searched)

#80 Overweight:ti,ab,kw (Word variations have been searched)

#81 MeSH descriptor: [Body Mass Index] explode all trees

#82 BMI:ti,ab,kw (Word variations have been searched)

#83 MeSH descriptor: [Cardiovascular Diseases] explode all trees

#84 Cardiovascular:ti,ab,kw (Word variations have been searched)

#85 CVD:ti,ab,kw (Word variations have been searched)

#86 Heart Disease*:ti,ab,kw (Word variations have been searched)

#87 MeSH descriptor: [Dental Caries] explode all trees

#88 Dental Decay:ti,ab,kw (Word variations have been searched)

#89 Caries:ti,ab,kw (Word variations have been searched)

#90 MeSH descriptor: [Neoplasms] explode all trees

#91 Neoplas*:ti,ab,kw (Word variations have been searched)

#92 Cancer:ti,ab,kw (Word variations have been searched)

#93 Tumor*:ti,ab,kw (Word variations have been searched)

#94 Tumour*:ti,ab,kw (Word variations have been searched)

#95 Carcinom*:ti,ab,kw (Word variations have been searched)

#96 Malignan*:ti,ab,kw (Word variations have been searched)

#97 MeSH descriptor: [Hypertension] explode all trees

#98 Hypertens*:ti,ab,kw (Word variations have been searched)

#99 Blood Pressure:ti,ab,kw (Word variations have been searched)

#100 #75 or #76 or #77 or #78 or #79 or #80 or #81 or #82 or #83 or #84 or #85 or #86 or #87 or #88 or #89 or #90 or #91 or #92 or #93 or #94 or #95 or #96 or #97 or #98 or #99

#101 #39 and #74 and #100

**EMBase (Elsevier)**

No. Query

#94  #30 AND #65 AND #93

#93  #66 OR #67 OR #68 OR #69 OR #70 OR #71 OR #72 OR #73 OR #74 OR #75 OR #76 OR #77 OR #78 OR #79 OR #80 OR #81 OR #82 OR #83 OR #84 OR #85 OR #86 OR #87 OR #88 OR #89 OR #90 OR #91 OR #92

#92  'blood pressure':ti,ab

#91  hypertens*:ti,ab

#90  'hypertension'/exp

#89  malignan*:ti,ab

#88  carcinom*:ti,ab

#87  tumour*:ti,ab

#86  tumor*:ti,ab

#85  cancer:ti,ab

#84  neoplas*:ti,ab

#83  'neoplasm'/exp

#82  caries:ti,ab

#81  'dental decay':ti,ab

#80  'dental caries'/exp

#79  'heart diseases':ti,ab

#78  'heart disease':ti,ab

#77  'cvd':ti,ab

#76  'cardiovascular':ti,ab

#75  'cardiovascular disease'/exp

#74  'body mass':ti,ab

#73  bmi:ti,ab

#72  'body mass'/exp

#71  overweight:ti,ab

#70  obes*:ti,ab

#69  'obesity'/exp

#68  diabetic*:ti,ab

#67  diabetes:ti,ab

#66  'diabetes mellitus'/exp

#65  #54 OR #64

#64  #60 AND #63

#63  #61 OR #62

#62  drink*:ti,ab

#61  beverage*:ti,ab

#60  #55 OR #56 OR #57 OR #58 OR #59

#59  fructose*:ti,ab

#58  sucrose*:ti,ab

#57  syrupe*:ti,ab

#56  'sugar intake'/exp

#55  'sweetening agent'/exp

#54  #31 OR #32 OR #33 OR #34 OR #35 OR #36 OR #37 OR #38 OR #39 OR #40 OR #41 OR #42 OR #43 OR #44

OR #45 OR #46 OR #47 OR #48 OR #49 OR #50 OR #51 OR #52 OR #53

#53  'fizzy beverage':ti,ab OR 'fizzy beverages':ti,ab

#52  'fizzy drink':ti,ab OR 'fizzy drinks':ti,ab

#51  juice*:ti,ab

#50  'fruit and vegetable juice'/exp

#49  'fruit beverage':ti,ab OR 'fruit beverages':ti,ab

#48  'fruit drink':ti,ab OR 'fruit drinks':ti,ab

#47  'sport beverage':ti,ab OR 'sport beverages':ti,ab

#46  'sport drink':ti,ab OR 'sport drinks':ti,ab

#45  cola:ti,ab

#44  'sugary beverage':ti,ab OR 'sugary beverages':ti,ab

#43  'sugary drink':ti,ab OR 'sugary drinks':ti,ab

#42  'sugared beverage':ti,ab OR 'sugared beverages':ti,ab

#41  'sugared drink':ti,ab OR 'sugared drinks':ti,ab

#40  'sweetened beverage':ti,ab OR 'sweetened beverages':ti,ab

#39  'sweetened drink':ti,ab OR 'sweetened drinks':ti,ab

#38  'soft beverage':ti,ab OR 'soft beverages':ti,ab

#37  'soft drink':ti,ab OR 'soft drinks':ti,ab

#36  'carbonated drink':ti,ab OR 'carbonated drinks':ti,ab

#35  ssb:ti,ab

#34  'carbonated dirnk':ti,ab OR 'carbonated drinks':ti,ab

#33  'carbonated beverage':ti,ab OR 'carbonated beverages':ti,ab

#32  soda*:ti,ab

#31  'carbonated beverage'/exp

#30  #28 OR #29

#29  ((burden OR attributable* OR societal) NEAR/3 (model OR estimat* OR projection* OR projecting)):ti,ab

#28  #1 OR #2 OR #3 OR #4 OR #5 OR #6 OR #7 OR #8 OR #9 OR #10 OR #11 OR #12 OR #13 OR #14 OR #15 OR

#16 OR #17 OR #18 OR #19 OR #20 OR #21 OR #22 OR #23 OR #24 OR #25 OR #26 OR #27

#27  'probabilistic model':ti,ab

#26  'statistical model':ti,ab

#25  'statistical model'/exp

#24  'regression analysis':ti,ab

#23  'regression analysis'/exp

#22  'monte carlo':ti,ab

#21  'monte carlo method'/exp

#20  markov:ti,ab

#19  'markov chain'/exp

#18  'policy evaluation':ti,ab

#17  'modelling':ti,ab

#16  'public health intervention':ti,ab

#15  'public health model':ti,ab

#14  'mathematical analysis':ti,ab

#13  'mathematical model':ti,ab

#12  'policy model':ti,ab

#11  qaly*:ti,ab

#10  daly*:ti,ab

#9  'risk assessment':ti,ab

#8  'comparative risk':ti,ab

#7  cost NEAR/1 effectiv*

#6  'cost benefit':ti,ab

#5  'cost benefit analysis'/exp

#4  'decision analysis':ti,ab

#3  'econometric model':ti,ab OR 'econometric models':ti,ab

#2  'economic model':ti,ab OR 'economic models':ti,ab

#1  'economic model'/exp

**CINAHL (EBSCO)**

# Query

S99 S36 AND S71 AND S98

S98 S72 OR S73 OR S74 OR S75 OR S76 OR S77 OR S78 OR S79 OR S80 OR S81 OR S82 OR S83 OR S84 OR S85 OR S86 OR

S87 OR S88 OR S89 OR S90 OR S91 OR S92 OR S93 OR S94 OR S95 OR S96 OR S97

S97 TI Blood Pressure OR AB Blood Pressure

S96 TI Hypertens* OR AB Hypertens*

S95 (MH "Hypertension+")

S94 TI Malignan* OR AB Malignan*

S93 TI Carcinom* OR AB Carcinom*

S92 TI Tumour* OR AB Tumour*

S91 TI Tumor* OR AB Tumor*

S90 TI Cancer OR AB Cancer

S89 TI Neoplas* OR AB Neoplas*

S88 (MH "Neoplasms+")

S87 TI Caries OR AB Caries

S86 TI Dental Decay OR AB Dental Decay

S85 (MH "Dental Caries")

S84 TI Heart Disease* OR AB Heart Disease*

S83 TI CVD OR AB CVD

S82 TI Cardiovascular OR AB Cardiovascular

S81 (MH "Cardiovascular Diseases+")

S80 TI Body Mass OR AB Body Mass

S79 TI BMI OR AB BMI

S78 (MM "Body Mass Index")

S77 TI Overweight OR AB Overweight

S76 TI Obes* OR AB Obes*

S75 (MH "Obesity+")

S74 TI Diabetic* OR AB Diabetic*

S73 TI Diabetes OR AB Diabetes

S72 (MH "Diabetes Mellitus+")

S71 S60 OR S70

S70 S66 AND S69

S69 S67 OR S68

S68 TI Drink* OR AB Drink*

S67 TI Beverage* OR AB Beverage*

S66 S61 OR S62 OR S63 OR S64 OR S65

S65 TI Fructose* OR AB Fructose*

S64 TI Sucrose OR AB Sucrose

S63 TI Syrupe* OR AB Syrupe*

S62 (MH "Dietary Sucrose")

S61 (MH "Sweetening Agents+")

S60 S37 OR S38 OR S39 OR S40 OR S41 OR S42 OR S43 OR S44 OR S45 OR S46 OR S47 OR S48 OR S49 OR S50 OR S51 OR S52 OR S53 OR S54 OR S55 OR S56 OR S57 OR S58 OR S59

S59 TI Fizzy Beverage* OR AB Fizzy Beverage*

S58 TI Fizzy Drink* OR AB Fizzy Drink*

S57 TI Juice* OR AB Juice*

S56 (MH "Fruit Juices+")

S55 TI Fruit Beverage* OR AB Fruit Beverage*

S54 TI Fruit Drink* OR AB Fruit Drink*

S53 TI Sport Beverage* OR AB Sport Beverage*

S52 TI Sport Drink* OR AB Sport Drink*

S51 TI Cola OR AB Cola

S50 TI Sugary Beverage* OR AB Sugary Beverage*

S49 TI Sugary Drink* OR AB Sugary Drink*

S48 TI Sugared Beverage* OR AB Sugared Beverage*

S47 TI Sugared Drink* OR AB Sugared Drink*

S46 TI Sweetened Beverage* OR AB Sweetened Beverage*

S45 TI Sweetened Drink* OR AB Sweetened Drink*

S44 TI Soft Beverage* OR AB Soft Beverage*

S43 TI Soft Drink* OR AB Soft Drink*

S42 TI Carbonated Drink* OR AB Carbonated Drink*

S41 TI SSB OR AB SSB

S40 TI Carbonated Drink* OR AB Carbonated Drink*

S39 TI Carbonated Beverage* OR AB Carbonated Beverage*

S38 TI Soda* OR AB Soda*

S37 (MH "Carbonated Beverages")

S36 S25 OR S35

S35 S29 AND S34

S34 S30 OR S31 OR S32 OR S33

S33 TI Projecting OR AB Projecting

S32 TI Projection* OR AB Projection*

S31 TI Estimat* OR AB Estimat*

S30 TI Model* OR AB Model*

S29 (S26 OR S27 OR S28)

S28 TI Societal Cost* OR AB Societal Cost*

S27 TI Attributable* OR AB Attributable*

S26 TI Burden OR AB Burden

S25 S1 OR S2 OR S3 OR S4 OR S5 OR S6 OR S7 OR S8 OR S9 OR S10 OR S11 OR S12 OR S13 OR S14 OR S15 OR S16 OR

S17 OR S18 OR S19 OR S20 OR S21 OR S22 OR S23 OR S24

S24 TI Probabilistic Model* OR AB Probabilistic Model*

S23 TI Statistical Model* OR AB Statistical Model*

S22 (MH "Models, Statistical")

S21 TI Regression Analy* OR AB Regression Analy*

S20 (MM "Regression")

S19 TI Monte Carlo OR AB Monte Carlo

S18 TI Markov OR AB Markov

S17 TI Policy Evaluation* OR AB Policy Evaluation*

S16 TI Modelling OR AB Modelling

S15 TI Public Health Intervention* OR AB Public Health Intervention*

S14 TI public health model OR AB Public Health Model*

S13 TI Mathematical Analys* OR AB Mathematical Analys*

S12 TI Mathematical Model* OR AB Mathematical Model*

S11 TI Policy Model OR AB Policy Model

S10 TI QALY* OR AB QALY*

S9 TI DALY* OR AB DALY*

S8 TI Risk Assess* OR AB Risk Assess*

S7 TI Comparative Risk OR AB Comparative Risk

S6 TI Cost Effectiv* OR AB Cost Effectiv*

S5 TI Cost Benefit OR AB Cost Benefit

S4 (MM "Cost Benefit Analysis")

S3 TI Decision Analy* OR AB Decision Analy*

S2 TI Econometric Model* OR AB Econometric Model*

S1 TI Economic Model* OR AB Economic Model*

**LILACS iAH**

(MH Modelos Económicos OR MH Análisis Costo-Beneficio OR (Econom$ AND Model$) OR ((Decision OR Decisão) AND Analis$)) OR ((Comparati$ OR Assess$ Evalua$) AND (Risk OR Riesgo$ OR Risco$)) OR DALY$ OR QALY$ OR ((Model$ OR Interven$) AND (Policy OR Public$))) AND (((Burden OR Carga OR Attributable OR Atribui$) AND (Model$)) AND (MH Carbonated Beverages OR Soda OR Refrigerante$ OR Refresco$ OR ((Bebida$ OR Drink$ OR Beverage$) AND (Carbonat$ OR Gaseosa$ OR Soft OR Azucarad$ OR Sugar$ OR Fruit OR Fruta$ OR Endulzad$ OR Adoçad$ OR Sport OR Deportiv$ OR Esportiv$ OR Sucros$ OR Syrupe OR Jarabe OR Xarope OR Fructos$ OR Fizzy OR Efervescente)) OR Juice$ OR Jugo$ OR Suco$ OR Cola) [Palabras]
