# Supplementary material for: Sugar sweetened beverages attributable disease burden and the potential impact of policy interventions: a systematic review of epidemiological and decision models
Source: BMC Public Health. 2021 Jul 27;21:1460. doi: 10.1186/s12889-021-11046-7 (PMC8317409; doi:10.1186/s12889-021-11046-7)
Supplement: Supplementary file 2 — Additional file 2. Complementary tables [file 12889_2021_11046_MOESM2_ESM.docx]

**Sugar sweetened beverages attributable disease burden and the potential impact of policy interventions: a systematic review of epidemiological and decision models.**

**Additional file 2. Complementary tables.**

**Table 1. Model features by study**

| **Study ID** | **Type / Name** | **Sugar-specific** | **Time horizon** | **Population** | **Country/region** | **Perspectives** |
| --- | --- | --- | --- | --- | --- | --- |
| Afshin 2015[[1](#_ENREF_1)] | Comparative risk assessment framework | No | 1 year | Adults (>=22 years) | Middle east and north Africa | Health system |
| Barrientos-G. 2017[[2](#_ENREF_2)] | Markov cohort, based on a model of diabetes incidence | Yes | 10 years | Adults (>=20 years) | Mexico | Health system |
| Basu 2013[[3](#_ENREF_3)] | Stochastic, discrete-time microsimulation model (6.5 million children attending childcare facilities) | No | 10 years | Adults | United States of America | Government |
| Basu 2014a[[4](#_ENREF_4)] | Stochastic, discrete-time microsimulation model | Yes | 10 years | Adults | India | Government |
| Basu 2014b[[5](#_ENREF_5)] | Stochastic, discrete-time, agent-based mathematical model + cost-effectiveness analysis | Yes | 20 years | Adults | United States of America | Government |
| Breeze 2017[[6](#_ENREF_6)] | Individual level simulation | No | Lifetime | Adults (>=16 years) | United Kingdom | Societal |
| Briggs 2013a[[7](#_ENREF_7)] | Comparative risk assessment framework | Yes | 1 year | Adults | Ireland | Health system |
| Briggs 2013b[[8](#_ENREF_8)] | Econometric and comparative risk assessment | Yes | 1 year | Adults (>=20 years) | United Kingdom | Government |
| Briggs 2017[[9](#_ENREF_9)] | Comparative risk assessment framework | Yes | 1 year | Total population | United Kingdom | Health system |
| Brown 2018[[10](#_ENREF_10)] | Proportional multi-state lifetable model | No | 100 years | Childs (5 to 15 years) | Australia | Societal |
| Cobiac 2017[[11](#_ENREF_11)] | Proportional multi-state lifetable model | No | Lifetime | Total population | Australia | Health system |
| Collins 2015[[12](#_ENREF_12)] | Montecarlo simulation | Yes | 20 years | Total population | United Kingdom | Health system |
| Crino 2017[[13](#_ENREF_13)] | Proportional multi-state lifetable model (Obesity Model) | No | Lifetime | Total population (2 to 100 years) | Australia | Societal |
| Gortmaker 2015a[[14](#_ENREF_14)] | Markov cohort CHOICES-ACE (based on ACE Prevention, but modified with U.S. Population and healthcare costs) | No | 10 years, lifetime | Total population | United States of America | Societal |
| Gortmaker 2015b[[15](#_ENREF_15)] | Microsimulation model based on ACE Prevention (modified with U.S. population, healthcare costs, morbidity, and mortality data) | No | 10 years | Total population | United States of America | Societal |
| Kristensen 2014[[16](#_ENREF_16)] | Microsimulation | No | 1 year | Childs (6 to 18 years) | United States of America | Health system |
| Lal 2017[[17](#_ENREF_17)] | Proportional multi-state lifetable model (Obesity Model) | No | Lifetime | Total population (2 to 100 years) | Australia | Societal |
| Lee 2018[[18](#_ENREF_18)] | Agent based model | No | 7 years | Childs (11 to 18 years) | United States of America | Health system |
| Lieffers 2018[[19](#_ENREF_19)] | Population attributable fraction based | No | 1 year | Total population (>=2 years) | Canada | Health system |
| Lin 2011[[20](#_ENREF_20)] | Dynamic population-based model | Yes | 10 years | Adults | United States of America | Government |
| Long 2015[[21](#_ENREF_21)] | Markov cohort (Obesity Model) | Yes | 10 years | Total population (2 to 100 years) | United States of America | Societal |
| Ma 2016[[22](#_ENREF_22)] | Dynamic population-based model | Yes | 5 years | Adults | United Kingdom | Government |
| Magnus 2016[[23](#_ENREF_23)] | Proportional multi-state lifetable model | No | Lifetime | Australian Aboriginal and Torres Strait Islander population | Australia | Societal |
| Manyema 2014[[24](#_ENREF_24)] | Montecarlo simulation | Yes | 1 year | Adults (>=20 years) | South Africa | Health system |
| Manyema 2015[[25](#_ENREF_25)] | Life table-based model in Microsoft Excel - Montecarlo simulation | Yes | 20 years | Adults (>=20 years) | South Africa | Health system |
| Manyema 2016[[26](#_ENREF_26)] | Life table-based model in Microsoft Excel - Montecarlo simulation | Yes | 20 years | Adults (>=20 years) | South Africa | Health system |
| Mekonnen 2013[[27](#_ENREF_27)] | Dynamic population-based model | No | 10 years | Adults | United States of America | Health system |
| Nomaguchi 2017[[28](#_ENREF_28)] | Proportional multi-state lifetable model (Obesity Model) | Yes | Lifetime | Adults (>=20 years) | Australia | Societal |
| Pearson-Stuttard 2017[[29](#_ENREF_29)] | Model adaptations | No | 15 years | Adults (>=25 years) | United States of America | Health system |
| Penalvo 2017[[30](#_ENREF_30)] | Comparative risk assessment framework | No | 1 year | Adults (>=25 years) | United States of America | Health system |
| Rezende 2016[[31](#_ENREF_31)] | Population attributable fraction based | No | 20 years | Total population | Brazil | Health system |
| Sacks 2011[[32](#_ENREF_32)] | Markov | No | 10 years | Adults (>=20 years) | Australia | Health system |
| Sanchez-R. 2016[[33](#_ENREF_33)] | Dynamic population-based model | No | 10 years | Adults (35 to 94 years) | Mexico | Health system |
| Schwendicke 2016[[34](#_ENREF_34)] | Microsimulation | Yes | 10 years | Adolescents and adults (14 to 79 years) | Germany | Health system |
| Singh 2015[[35](#_ENREF_35)] | Comparative risk assessment framework | Yes | 1 year | Adults (>=20 years) | Worldwide | Health system |
| Vecino-Ortiz 2018[[36](#_ENREF_36)] | Comparative risk assessment framework | Yes | Lifetime | Total population | Colombia | Public Health sector |
| Veerman 2016[[37](#_ENREF_37)] | Proportional multi-state lifetable model | Yes | 25 years | Total population | Australia | Societal |
| Wang 2012[[38](#_ENREF_38)] | Dynamic population-based model | No | 10 years | Adults | United States of America | Health system |
| Wilson 2015[[39](#_ENREF_39)] | Physiology, population, healthcare system, and outcomes models | No | 5, 10, 20 years | Argely Hispanic population residing along the United States -Mexico border. | United States of America | Societal |
| Wright 2015[[40](#_ENREF_40)] | Markov cohort CHOICES-ACE | No | 10 years | Preschool-aged children attending childcare facilities | United States of America | Health system (statal level) |

CHOICES: Childhood Obesity Intervention Cost-Effectiveness Study. ACE: Assessing cost-effectiveness.

**Table 2. Model inputs by study**

| **Study ID** | **Incidence** | **Vital statistics** | **Longitudinal data** | **Representative survey** | **Other** |
| --- | --- | --- | --- | --- | --- |
| Afshin 2015[[1](#_ENREF_1)] | No | Yes | No | Yes | Relative Risk (RR) for consumption of SSB and health conditions |
| Barrientos-G. 2017[[2](#_ENREF_2)] | Yes | Yes | Yes | Yes | Elasticity/consumption |
| Basu 2013[[3](#_ENREF_3)] | No | No | No | No | Elasticity/consumption |
| Basu 2014a[[4](#_ENREF_4)] | No | No | No | No | Elasticity/purchases |
| Basu 2014b[[5](#_ENREF_5)] | No | No | No | No | Elasticity/consumption |
| Breeze 2017[[6](#_ENREF_6)] | Yes | Yes | Yes | Yes | No |
| Briggs 2013a[[7](#_ENREF_7)] | No | No | No | Yes | No |
| Briggs 2013b[[8](#_ENREF_8)] | No | No | No | Yes | SSB price, expense and quantities purchased |
| Briggs 2017[[9](#_ENREF_9)] | Yes | No | Yes | Yes | Sales, consumption and elasticity. |
| Brown 2018[[10](#_ENREF_10)] | Yes | Yes | No | Yes | Nine obesity-related diseases (ischemic heart disease, hypertensive heart disease, ischemic stroke, diabetes, colorectal cancer, kidney cancer, breast cancer, endometrial cancer and osteoarthritis). |
| Cobiac 2017[[11](#_ENREF_11)] | Yes | Yes | No | Yes | Lethality and costs |
| Collins 2015[[12](#_ENREF_12)] | No | Yes | No | Yes | No |
| Crino 2017[[13](#_ENREF_13)] | Yes | Yes | No | Yes | No |
| Gortmaker 2015a[[14](#_ENREF_14)] | Yes | Yes | No | Yes | No |
| Gortmaker 2015b[[15](#_ENREF_15)] | Yes | Yes | No | Yes | No |
| Kristensen 2014[[16](#_ENREF_16)] | No | No | Yes | No | No |
| Lal 2017[[17](#_ENREF_17)] | Yes | Yes | No | Yes | No |
| Lee 2018[[18](#_ENREF_18)] | No | No | Yes | Yes | Geographic data of population distribution, schools, kiosks, greengrocers, purchasing and consumption patterns |
| Lieffers 2018[[19](#_ENREF_19)] | Yes | Yes | No | Yes | No |
| Lin 2011[[20](#_ENREF_20)] | No | No | No | Yes | No |
| Long 2015[[21](#_ENREF_21)] | Yes | Yes | No | Yes | No |
| Ma 2016[[22](#_ENREF_22)] | No | No |  |  | National Diet Survey and Soft Drinks Association annual reports |
| Magnus 2016[[23](#_ENREF_23)] | Yes | Yes | No | Yes | Consumption of sodium in foods |
| Manyema 2014[[24](#_ENREF_24)] | No | Yes | No | Yes | Elasticity |
| Manyema 2015[[25](#_ENREF_25)] | No | Yes | No | Yes | Elasticity |
| Manyema 2016[[26](#_ENREF_26)] | No | Yes | No | Yes | Elasticity |
| Mekonnen 2013[[27](#_ENREF_27)] | Yes | Yes | Yes | Yes | Elasticity/consumption |
| Nomaguchi 2017[[28](#_ENREF_28)] | Yes | Yes | No | Yes | Productivity |
| Pearson-Stuttard 2017[[29](#_ENREF_29)] | Yes | Yes | No | Yes | Consumption of fruits, vegetables, SSB, by age and sex. |
| Penalvo 2017[[30](#_ENREF_30)] | No | Yes | No | Yes | No |
| Rezende 2016[[31](#_ENREF_31)] | No | Yes | Yes | Yes | No |
| Sacks 2011[[32](#_ENREF_32)] | Yes | Yes | No | Yes | No |
| Sanchez-R. 2016[[33](#_ENREF_33)] | Yes | Yes | Yes | Yes | Elasticity/consumption |
| Schwendicke 2016[[34](#_ENREF_34)] | Yes | No | No | No | No |
| Singh 2015[[35](#_ENREF_35)] | No | Yes | No | Yes | DALYs del GBD, RR for consumption de SSB, diabetes, BMI and other conditions |
| Vecino-Ortiz 2018[[36](#_ENREF_36)] | No | Yes | No | Yes | Socio-economic level surveys and SSBs baseline consumption data |
| Veerman 2016[[37](#_ENREF_37)] | Yes | No | No | Yes | No |
| Wang 2012[[38](#_ENREF_38)] | Yes | Yes | Yes | Yes | Elasticity/consumption |
| Wilson 2015[[39](#_ENREF_39)] | Yes | Yes | No | Yes | Anatomic and biologic variables for the Archimedes model |
| Wright 2015[[40](#_ENREF_40)] | No | No | No | Yes | No |

SSB: sugar-sweetened beverage. DALY: Disability Adjusted Life Years. GBD: Global Burden of Disease. BMI: Body Mass Index.

**Table 3. Model outcomes 1, by study**

| **Study ID** | **Variation in consumption** | **Incidence** | **Prevalence** | **Mortality** | **Life years** | **DALYs/**  **QALYs** | **Direct**  **costs** | **Indirect costs** | **Tax collection** | **SSBs Sales** |
| --- | --- | --- | --- | --- | --- | --- | --- | --- | --- | --- |
| Afshin 2015[[1](#_ENREF_1)] | No | No | No | Yes | No | No | No | No | No | No |
| Barrientos-G. 2017[[2](#_ENREF_2)] | Yes | No | No | No | No | No | No | No | No | No |
| Basu 2013[[3](#_ENREF_3)] | Yes | No | No | No | No | No | No | No | No | Yes |
| Basu 2014a[[4](#_ENREF_4)] | Yes | No | No | No | No | No | No | No | No | Yes |
| Basu 2014b[[5](#_ENREF_5)] | Yes | No | No | No | No | No | No | No | No | Yes |
| Breeze 2017[[6](#_ENREF_6)] | Yes | No | No | Yes | Yes | Yes | Yes | Yes | No | No |
| Briggs 2013a[[7](#_ENREF_7)] | Yes | No | Yes | No | No | No | No | No | No | No |
| Briggs 2013b[[8](#_ENREF_8)] | Yes | No | Yes | No | No | No | No | No | Yes | No |
| Briggs 2017[[9](#_ENREF_9)] | No | Yes | Yes | No | No | No | No | No | No | No |
| Brown 2018[[10](#_ENREF_10)] | Yes | No | No | Yes | Yes | Yes | Yes | No | No | No |
| Cobiac 2017[[11](#_ENREF_11)] | Yes | No | No | No | No | Yes | Yes | No | No | No |
| Collins 2015[[12](#_ENREF_12)] | Yes | No | No | No |  | Yes | Yes | No | No | No |
| Crino 2017[[13](#_ENREF_13)] | Yes | No | No | Yes | Yes | Yes | Yes | No | No | No |
| Gortmaker 2015a[[14](#_ENREF_14)] | Yes | No | No | No | Yes | Yes | Yes | Yes | No | No |
| Gortmaker 2015b[[15](#_ENREF_15)] | Yes | No | No | No | No | No | Yes | Yes | No | No |
| Kristensen 2014[[16](#_ENREF_16)] | Yes | No | No | No | No | No | No | No | No | No |
| Lal 2017[[17](#_ENREF_17)] | Yes | No | No | Yes | Yes | Yes | Yes | No | Yes | Yes |
| Lee 2018[[18](#_ENREF_18)] | Yes | No | No | No | No | No | No | No | No | No |
| Lieffers 2018[[19](#_ENREF_19)] | Yes | No | No | Yes | No | No | Yes | Yes | No | No |
| Lin 2011[[20](#_ENREF_20)] | Yes | Yes | No | No | No | No | No | No | Yes | Yes |
| Long 2015[[21](#_ENREF_21)] | Yes | No | No | Yes | Yes | Yes | Yes | No | Yes | No |
| Ma 2016[[22](#_ENREF_22)] | Yes | No | Yes | No | No | No | No | No | No | No |
| Magnus 2016[[23](#_ENREF_23)] | Yes | No | No | Yes | Yes | Yes | Yes | No | No | No |
| Manyema 2014[[24](#_ENREF_24)] | Yes | No | Yes | No | No | No | No | No | No | No |
| Manyema 2015[[25](#_ENREF_25)] | Yes | No | Yes | Yes | Yes | Yes | Yes | No | No | No |
| Manyema 2016[[26](#_ENREF_26)] | Yes | Yes | No | Yes | No | Yes | Yes | No | No | No |
| Mekonnen 2013[[27](#_ENREF_27)] | Yes | No | No | Yes | No | No | Yes | No | No | No |
| Nomaguchi 2017[[28](#_ENREF_28)] | Yes | No | No | Yes | Yes | Yes | Yes | Yes | Yes | Yes |
| Pearson-Stuttard 2017[[29](#_ENREF_29)] | Yes | No | No | Yes | No | No | No | No | No | No |
| Penalvo 2017[[30](#_ENREF_30)] | No | No | No | Yes | No | No | No | No | No | No |
| Rezende 2016[[31](#_ENREF_31)] | Yes | No | No | Yes | No | No | No | No | No | No |
| Sacks 2011[[32](#_ENREF_32)] | Yes | No | No | No | No | Yes | Yes | No | No | No |
| Sanchez-R. 2016[[33](#_ENREF_33)] | Yes | No | No | Yes | No | No | Yes | No | No | No |
| Schwendicke 2016[[34](#_ENREF_34)] | Yes | No | No | No | No | No | Yes | No | Yes | No |
| Singh 2015[[35](#_ENREF_35)] | No | No | No | Yes | No | Yes | No | No | No | No |
| Vecino-Ortiz 2018[[36](#_ENREF_36)] |  | No | No | No | No | No | No | No | No | No |
| Veerman 2016[[37](#_ENREF_37)] | Yes | No | No | Yes | Yes | Yes | Yes | No | Yes | Yes |
| Wang 2012[[38](#_ENREF_38)] | Yes | No | No | Yes | No | No | Yes | No | No | No |
| Wilson 2015[[39](#_ENREF_39)] | No | No | No | Yes | Yes | Yes | Yes | Yes | No | No |
| Wright 2015[[40](#_ENREF_40)] | Yes | No | No | No | No | No | Yes | No | No | Yes |

SSBs: sugar-sweetened beverages.

**Table 4. Model outcomes 2, by study**

| **Study ID** | **Equity** | **Obesity/**  **Overweight** | **Diabetes** | **Cardiovascular disease** | **Cancer** | **Cavities** | **Bulling** | **Other** |
| --- | --- | --- | --- | --- | --- | --- | --- | --- |
| Afshin 2015[[1](#_ENREF_1)] | No | Aggregated | Aggregated | Aggregated | No | No | No | No |
| Barrientos-G. 2017[[2](#_ENREF_2)] | No | Yes | Yes | No | No | No | No | No |
| Basu 2013[[3](#_ENREF_3)] | Yes | Yes | Yes | No | No | No | No | Incremental costs, quality-adjusted life-years (QALYs), body mass index, Alternative Healthy Eating Index, Food Security Score, diabetes person-years, and deaths from myocardial infarctions and strokes. |
| Basu 2014a[[4](#_ENREF_4)] | Yes | Yes | Yes | No | No | No | No | Per capita kilocalorie and glycemic load changes |
| Basu 2014b[[5](#_ENREF_5)] | Yes | Yes | Yes | No | No | No | No | Per capita kilocalorie and glycemic load changes |
| Breeze 2017[[6](#_ENREF_6)] | No | Aggregated | Yes | Yes | Yes | No | No | Depression and diabetes related conditions (blindness, ulcer, renal insufficiency, amputation) |
| Briggs 2013a[[7](#_ENREF_7)] | Yes | Yes | No | No | No | No | No | No |
| Briggs 2013b[[8](#_ENREF_8)] | Yes | Yes | No | No | No | No | No | SSBs expenditure |
| Briggs 2017[[9](#_ENREF_9)] | No | Yes | Yes | No | No | Yes | No | No |
| Brown 2018[[10](#_ENREF_10)] | Yes | Yes | Aggregated | Aggregated | Yes | No | No | No |
| Cobiac 2017[[11](#_ENREF_11)] | No | No | No | Yes | No | No | No | No |
| Collins 2015[[12](#_ENREF_12)] | No | No | Yes | Yes | Yes | No | No | No |
| Crino 2017[[13](#_ENREF_13)] | No | Yes | Aggregated | Aggregated | Aggregated | No | No | Legislation costs, industry costs |
| Gortmaker 2015a[[14](#_ENREF_14)] | Yes | Yes | Yes | Yes | Yes | No | No | Arthritis |
| Gortmaker 2015b[[15](#_ENREF_15)] | No | Yes | Yes | Yes | Yes | No | No | No |
| Kristensen 2014[[16](#_ENREF_16)] | No | Yes | No | No | No | No | No | No |
| Lal 2017[[17](#_ENREF_17)] | Yes | Yes | Aggregated | Aggregated | Aggregated | No | No | Elasticity, legislation costs, implementing tax, out of pocket costs; returns on investment in healthcare cost, savings in 10 years, concentration index |
| Lee 2018[[18](#_ENREF_18)] | No | Yes | No | No | No | No | No | No |
| Lieffers 2018[[19](#_ENREF_19)] | No | Aggregated | Yes | Yes | Yes | No | No | No |
| Lin 2011[[20](#_ENREF_20)] | Yes | Yes | No | No | No | No | No | No |
| Long 2015[[21](#_ENREF_21)] | Yes | Yes | Yes | Yes | Yes | No | No | BMI, stroke, ischemic heart disease, hypertensive heart disease, diabetes mellitus, osteoarthritis, post-menopausal breast cancer, colon cancer, endometrial cancer, and kidney cancer |
| Ma 2016[[22](#_ENREF_22)] | No | Yes | Aggregated | Yes | No | No | No | No |
| Magnus 2016[[23](#_ENREF_23)] | No | Aggregated | Aggregated | Aggregated | Aggregated | No | No | Societal costs |
| Manyema 2014[[24](#_ENREF_24)] | No | Yes | No | No | No | No | No | No |
| Manyema 2015[[25](#_ENREF_25)] | No | Yes | Yes | No | No | No | No | No |
| Manyema 2016[[26](#_ENREF_26)] | No | Yes | No | Yes | No | No | No | No |
| Mekonnen 2013[[27](#_ENREF_27)] | No | Yes | Yes | Yes | No | No | No | Blood pressure |
| Nomaguchi 2017[[28](#_ENREF_28)] | Yes/No | Yes | Yes | Yes | Yes | No | No | Osteoarthritis |
| Pearson-Stuttard 2017[[29](#_ENREF_29)] | Yes | No | No | Yes | No | No | No | No |
| Penalvo 2017[[30](#_ENREF_30)] | Yes | No | Yes | Yes | No | No | No | No |
| Rezende 2016[[31](#_ENREF_31)] | No | No | Yes | No | No | No | No | No |
| Sacks 2011[[32](#_ENREF_32)] | No | Yes | Yes | Yes | No | No | No | No |
| Sanchez-R. 2016[[33](#_ENREF_33)] | No | Yes | Aggregated | Yes | No | No | No | No |
| Schwendicke 2016[[34](#_ENREF_34)] | No | No | No | No | No | Yes | No | No |
| Singh 2015[[35](#_ENREF_35)] | No | Aggregated | Aggregated | Aggregated | Aggregated | No | No | No |
| Vecino-Ortiz 2018[[36](#_ENREF_36)] | No | No | No | No | No | No | No | No |
| Veerman 2016[[37](#_ENREF_37)] | No | Yes | Yes | Yes | Yes | No | No | Disease-related quality of life. BMI, stroke, ischemic heart disease, hypertensive heart disease, diabetes mellitus, osteoarthritis, post-menopausal breast cancer, colon cancer, endometrial cancer, and kidney cancer |
| Wang 2012[[38](#_ENREF_38)] | No | Yes | Yes | Yes | No | No | No | No |
| Wilson 2015[[39](#_ENREF_39)] | No | Yes | Aggregated | Aggregated |  |  |  | No |
| Wright 2015[[40](#_ENREF_40)] | Yes | Yes | No | No | No | No | No | METS, total balance energy, exposure time to screens |

BMI: Body Mass Index. METS: metabolic equivalents.

**Table 5. Model subgroups, interventions assessed and applicability by study**

| **Study ID** | **Subgroups** | | | | **interventions assessed** | | | | | **Applicability** | | |
| --- | --- | --- | --- | --- | --- | --- | --- | --- | --- | --- | --- | --- |
|  | **Children/teenage** | **Gender** | **Income level** | **Vulnerable group** | **Taxes** | **School environment** | **Adver-tising** | **Label-ling** | **Other** | **Effort / requirement** | **Applicability / reproducibility** |  |
| Afshin 2015[[1](#_ENREF_1)] | Yes | No | Yes | Socioeconomic level area | No | No | No | No | No | Moderate | Moderate |  |
| Barrientos-G. 2017[[2](#_ENREF_2)] | Yes | No | No | No | Yes | No | No | No | No | Moderate | Low |  |
| Basu 2013[[3](#_ENREF_3)] | No | Yes | No | No | Yes | No | No | No | No | Moderate | Moderate |  |
| Basu 2014a[[4](#_ENREF_4)] | No | Yes | Yes | No | Yes | No | No | No | No | Moderate | High |  |
| Basu 2014b[[5](#_ENREF_5)] | Yes | Yes | Yes | Ethnicity | Yes | No | No | No | No | Moderate | Moderate |  |
| Breeze 2017[[6](#_ENREF_6)] | Yes | Yes | Yes | Rural/Urban status | Yes | No | No | No | No | High | Low |  |
| Briggs 2013a[[7](#_ENREF_7)] | Yes | Yes | No | Ethnicity | Yes | No | No | No | No | Moderate | Low |  |
| Briggs 2013b[[8](#_ENREF_8)] | No | No | No | No | Yes | No | No | No | No | Moderate | Low |  |
| Briggs 2017[[9](#_ENREF_9)] | No | Yes | Yes | No | Yes | No | No | No | No | Moderate | Low |  |
| Brown 2018[[10](#_ENREF_10)] | No | No | Yes | No | No | No | Yes | No | No | Moderate | Moderate |  |
| Cobiac 2017[[11](#_ENREF_11)] | Yes | Yes | No | No | Yes | Yes | No | No | No | Low | Moderate |  |
| Collins 2015[[12](#_ENREF_12)] | No | No | No | No | No | No | No | No | 20% sugary drinks duty on local authorities | Moderate | Moderate |  |
| Crino 2017[[13](#_ENREF_13)] | Yes | Yes | No | No | No | No | No | No | Portion limit and product reformulation | Moderate | Moderate |  |
| Gortmaker 2015a[[14](#_ENREF_14)] | Yes | Yes | No | No | Yes | Yes | Yes | No | No | Moderate | Moderate |  |
| Gortmaker 2015b[[15](#_ENREF_15)] | Yes | No | No | No | Yes | Yes | Yes | No | No | Moderate | Moderate |  |
| Kristensen 2014[[16](#_ENREF_16)] | Yes | No | No | No | Yes | No | No | No | No | Moderate | Low |  |
| Lal 2017[[17](#_ENREF_17)] | Yes | No | No | Ethnicity | Yes | No | No | No | No | Moderate | Moderate |  |
| Lee 2018[[18](#_ENREF_18)] | No | No | Yes | Socio-economic deprivation quintiles area | No | No | No | Yes | No | High | Low |  |
| Lieffers 2018[[19](#_ENREF_19)] | Yes | Yes | No | Literacy, City of residence | No | No | No | No | Eat well vs Eat like today | Low | Low |  |
| Lin 2011[[20](#_ENREF_20)] | Yes | Yes | No | No | Yes | No | No | No | No | Moderate | Moderate |  |
| Long 2015[[21](#_ENREF_21)] | No | No | Yes | No | Yes | No | No | No | No | Moderate | High |  |
| Ma 2016[[22](#_ENREF_22)] | Yes | Yes | No | No | Yes | No | No | No | No | Moderate | Moderate |  |
| Magnus 2016[[23](#_ENREF_23)] | No | No | Yes | No | Yes | No | No | No | No |  |  |  |
| Manyema 2014[[24](#_ENREF_24)] | Yes | Yes | No | No | Yes | No | No | No | No | Moderate | Low |  |
| Manyema 2015[[25](#_ENREF_25)] | No | Yes | No | No | Yes | No | No | No | No | Moderate | Moderate |  |
| Manyema 2016[[26](#_ENREF_26)] | No | Yes | No | No | Yes | No | No | No | No | Moderate | Low |  |
| Mekonnen 2013[[27](#_ENREF_27)] | No | Yes | No | No | Yes | No | No | No | No | Moderate | Low |  |
| Nomaguchi 2017[[28](#_ENREF_28)] | No | Yes | Yes | No | Yes | No | No | No | No | Moderate | High |  |
| Pearson-Stuttard 2017[[29](#_ENREF_29)] | Yes | Yes | Yes | Ethnicity | Yes | No | Yes | No | Subisdies | Moderate | Low |  |
| Penalvo 2017[[30](#_ENREF_30)] | Yes | Yes | Yes | Rural/Urban status | Yes | No | No | No | Subsidies | Moderate | Low |  |
| Rezende 2016[[31](#_ENREF_31)] | Yes | Yes | No | Ethnicity | No | No | No | No | No | Low | Low |  |
| Sacks 2011[[32](#_ENREF_32)] | No | No | No | No | Yes | No | No | Yes | No | Moderate | Moderate |  |
| Sanchez-R. 2016[[33](#_ENREF_33)] | No | Yes | Yes | No | Yes | No | No | No | No | Moderate | Low |  |
| Schwendicke 2016[[34](#_ENREF_34)] | No | No | Yes | No | Yes | No | No | No | No | Moderate | Low |  |
| Singh 2015[[35](#_ENREF_35)] | Yes | Yes | No | No | No | No | No | No | No | Moderate | Moderate |  |
| Vecino-Ortiz 2018[[36](#_ENREF_36)] | Yes | No | Yes | Socio-economic level area |  | Yes | No | No | No | Low | Moderate |  |
| Veerman 2016[[37](#_ENREF_37)] | No | No | No | No | Yes | No | No | No | No | Moderate | High |  |
| Wang 2012[[38](#_ENREF_38)] | Yes | Yes | No | No | Yes | No | No | No | No | Moderate | Low |  |
| Wilson 2015[[39](#_ENREF_39)] | Yes | Yes | No | No | No | No | No | No | Community-Based Weight Control Intervention | Moderate | Moderate |  |
| Wright 2015[[40](#_ENREF_40)] | Yes | No | No | No | No | Yes | No | No | No | Low | High |  |

**Table 6. Taxonomy of model structures by study**

| **Study ID** | **Interaction allowed** | **Aggregate/ Individual/ econometric/ epidemiological** | **Untimed, Timed Continuous** | **Cohort,**  **Multi-cohort** |
| --- | --- | --- | --- | --- |
| Afshin 2015[[1](#_ENREF_1)] | No | Epidemiological | Untimed | NR |
| Barrientos-G. 2017[[2](#_ENREF_2)] | No | Aggregated | Timed | Cohort |
| Basu 2013[[3](#_ENREF_3)] | No | Individual | Continuous | Multi-cohort |
| Basu 2014a[[4](#_ENREF_4)] | No | Individual | Continuous | Multi-cohort |
| Basu 2014b[[5](#_ENREF_5)] | No | Individual | Continuous | Multi-cohort |
| Breeze 2017[[6](#_ENREF_6)] | No | Individual | Timed | Cohort |
| Briggs 2013a[[7](#_ENREF_7)] | No | Epidemiological | Untimed | NR |
| Briggs 2013b[[8](#_ENREF_8)] | No | Epidemiological | Untimed | NR |
| Briggs 2017[[9](#_ENREF_9)] | No | Epidemiological | Untimed | NR |
| Brown 2018[[10](#_ENREF_10)] | No | Aggregated | Timed | Multi-cohort |
| Cobiac 2017[[11](#_ENREF_11)] | No | Aggregated | Continuous | Cohort |
| Collins 2015[[12](#_ENREF_12)] | No | Aggregated | Untimed | Cohort |
| Crino 2017[[13](#_ENREF_13)] | No | Aggregated | Timed | Multi-cohort |
| Gortmaker 2015a[[14](#_ENREF_14)] | No | Individual | Timed | Cohort |
| Gortmaker 2015b[[15](#_ENREF_15)] | No | Individual | Timed | Cohort |
| Kristensen 2014[[16](#_ENREF_16)] | No | Individual | Timed | Cohort |
| Lal 2017[[17](#_ENREF_17)] | No | Aggregated | Timed | Multi-cohort |
| Lee 2018[[18](#_ENREF_18)] | No | Individual | Timed | Cohort |
| Lieffers 2018[[19](#_ENREF_19)] | No | Epidemiological | Untimed | Not applicable |
| Lin 2011[[20](#_ENREF_20)] | No | Econometric | Not applicable | Not applicable |
| Long 2015[[21](#_ENREF_21)] | No | Individual | Timed | Cohort |
| Ma 2016[[22](#_ENREF_22)] | No | Individual | Continuous | Multi-cohort |
| Magnus 2016[[23](#_ENREF_23)] | No | Aggregated | Timed | Cohort |
| Manyema 2014[[24](#_ENREF_24)] | No | Epidemiological | Untimed | Cohort |
| Manyema 2015[[25](#_ENREF_25)] | No | Aggregated | Untimed | Cohort |
| Manyema 2016[[26](#_ENREF_26)] | No | Aggregated | Untimed | Cohort |
| Mekonnen 2013[[27](#_ENREF_27)] | No | Aggregated | Timed | Cohort |
| Nomaguchi 2017[[28](#_ENREF_28)] | No | Individual | Timed | Cohort |
| Pearson-Stuttard 2017[[29](#_ENREF_29)] | No | Aggregated | Untimed | Cohort |
| Penalvo 2017[[30](#_ENREF_30)] | No | Aggregated | Untimed | Cohort |
| Rezende 2016[[31](#_ENREF_31)] | No | Epidemiological | Untimed | Cohort |
| Sacks 2011[[32](#_ENREF_32)] | No | Individual | Timed | Cohort |
| Sanchez-R. 2016[[33](#_ENREF_33)] | No | Individual | Timed | Cohort |
| Schwendicke 2016[[34](#_ENREF_34)] | No | Individual | Timed | Cohort |
| Singh 2015[[35](#_ENREF_35)] | No | Epidemiological | Untimed | Cohort |
| Vecino-Ortiz 2018[[36](#_ENREF_36)] | No | Aggregated | Timed | NR |
| Veerman 2016[[37](#_ENREF_37)] | No | Aggregated | Timed | Multi-cohort |
| Wang 2012[[38](#_ENREF_38)] | No | Individual | Timed | Cohort |
| Wilson 2015[[39](#_ENREF_39)] | No | Individual | Continuous | Cohort |
| Wright 2015[[40](#_ENREF_40)] | No | Individual | Timed | Cohort |

**References**

1. Afshin A, Micha R, Khatibzadeh S, Fahimi S, Shi P, Powles J, Singh G, Yakoob MY, Abdollahi M, Al-Hooti S *et al*: **The impact of dietary habits and metabolic risk factors on cardiovascular and diabetes mortality in countries of the Middle East and North Africa in 2010: A comparative risk assessment analysis**. *BMJ Open* 2015, **5**(5).

2. Barrientos-Gutierrez T, Zepeda-Tello R, Rodrigues ER, Colchero-Aragones A, Rojas-Martõnez R, Lazcano-Ponce E, Hernandez-Avila M, Rivera-Dommarco J, Meza R: **Expected population weight and diabetes impact of the 1-peso-per-litre tax to sugar sweetened beverages in Mexico**. *PLoS ONE* 2017, **12**(5).

3. Basu S, Seligman H, Bhattacharya J: **Nutritional policy changes in the supplemental nutrition assistance program: a microsimulation and cost-effectiveness analysis**. *Medical Decision Making* 2013, **33**(7):937-948.

4. Basu S, Vellakkal S, Agrawal S, Stuckler D, Popkin B, Ebrahim S: **Averting obesity and type 2 diabetes in India through sugar-sweetened beverage taxation: an economic-epidemiologic modeling study**. *PLoS Medicine* 2014, **11**(1):e1001582-e1001582.

5. Basu S, Lewis K: **Reducing Added Sugars in the Food Supply Through a Cap-and-Trade Approach**. *American Journal of Public Health* 2014, **104**(12):2432-2438.

6. Breeze PR, Thomas C, Squires H, Brennan A, Greaves C, Diggle P, Brunner E, Tabak A, Preston L, Chilcott J: **Cost-effectiveness of population-based, community, workplace and individual policies for diabetes prevention in the UK**. *Diabetic Medicine* 2017, **34**(8):1136-1144.

7. Briggs AD, Mytton OT, Kehlbacher A, Tiffin R, Rayner M, Scarborough P: **Overall and income specific effect on prevalence of overweight and obesity of 20% sugar sweetened drink tax in UK: econometric and comparative risk assessment modelling study**. *BMJ* 2013, **347**:f6189.

8. Briggs AD, Mytton OT, Madden D, O'Shea D, Rayner M, Scarborough P: **The potential impact on obesity of a 10% tax on sugar-sweetened beverages in Ireland, an effect assessment modelling study**. *BMC public health* 2013, **13**:860.

9. Briggs ADM, Mytton OT, Kehlbacher A, Tiffin R, Elhussein A, Rayner M, Jebb SA, Blakely T, Scarborough P: **Health impact assessment of the UK soft drinks industry levy: a comparative risk assessment modelling study**. *The Lancet Public Health* 2017, **2**(1):e15-e22.

10. Brown V, Ananthapavan J, Veerman L, Sacks G, Lal A, Peeters A, Backholer K, Moodie M: **The Potential Cost-Effectiveness and Equity Impacts of Restricting Television Advertising of Unhealthy Food and Beverages to Australian Children**. *Nutrients* 2018, **10**(5):N.PAG-N.PAG.

11. Cobiac LJ, Tam K, Veerman L, Blakely T: **Taxes and Subsidies for Improving Diet and Population Health in Australia: A Cost-Effectiveness Modelling Study**. *PLoS Medicine* 2017, **14**(2).

12. Collins B, Capewell S, O'Flaherty M, Timpson H, Razzaq A, Cheater S, Ireland R, Bromley H: **Modelling the Health Impact of an English Sugary Drinks Duty at National and Local Levels**. *PLoS ONE [Electronic Resource]* 2015, **10**(6):e0130770.

13. Crino M, Mantilla Herrera AM, Ananthapavan J, Wu JHY, Neal B, Yong Yi L, Miaobing Z, Lal A, Sacks G: **Modelled Cost-Effectiveness of a Package Size Cap and a Kilojoule Reduction Intervention to Reduce Energy Intake from Sugar-Sweetened Beverages in Australia**. *Nutrients* 2017, **9**(9):1-17.

14. Gortmaker SL, Long MW, Resch SC, Ward ZJ, Cradock AL, Barrett JL, Wright DR, Sonneville KR, Giles CM, Carter RC *et al*: **Cost Effectiveness of Childhood Obesity Interventions: Evidence and Methods for CHOICES**. *American Journal of Preventive Medicine* 2015, **49**(1):102-111.

15. Gortmaker SL, Wang YC, Long MW, Giles CM, Ward ZJ, Barrett JL, Kenney EL, Sonneville KR, Sadaf Afzal A, Resch SC *et al*: **Three Interventions That Reduce Childhood Obesity Are Projected To Save More Than They Cost To Implement**. *Health Affairs* 2015, **34**(11):1932-1939.

16. Kristensen AH, Flottemesch TJ, Maciosek MV, Jenson J, Barclay G, Ashe M, Sanchez EJ, Story M, Teutsch SM, Brownson RC: **Reducing childhood obesity through U.S. federal policy: a microsimulation analysis**. *American Journal of Preventive Medicine* 2014, **47**(5):604-612.

17. Lal A, Mantilla-Herrera AM, Veerman L, Backholer K, Sacks G, Moodie M, Siahpush M, Carter R, Peeters A: **Modelled health benefits of a sugar-sweetened beverage tax across different socioeconomic groups in Australia: A cost-effectiveness and equity analysis**. *PLoS Medicine* 2017, **14**(6):1-17.

18. Lee BY, Ferguson MC, Hertenstein DL, Adam A, Zenkov E, Wang PI, Wong MS, Gittelsohn J, Mui Y, Brown ST: **Simulating the Impact of Sugar-Sweetened Beverage Warning Labels in Three Cities**. *American Journal of Preventive Medicine* 2018, **54**(2):197-204.

19. Lieffers JRL, Ekwaru JP, Ohinmaa A, Veugelers PJ: **The economic burden of not meeting food recommendations in Canada: The cost of doing nothing**. *PLoS ONE* 2018, **13**(4).

20. Lin BH, Smith TA, Lee JY, Hall KD: **Measuring weight outcomes for obesity intervention strategies: The case of a sugar-sweetened beverage tax**. *Economics and Human Biology* 2011, **9**(4):329-341.

21. Long MW, Gortmaker SL, Ward ZJ, Resch SC, Moodie ML, Sacks G, Swinburn BA, Carter RC, Claire Wang Y: **Cost Effectiveness of a Sugar-Sweetened Beverage Excise Tax in the U.S**. *American Journal of Preventive Medicine* 2015, **49**(1):112-123.

22. Ma Y, He FJ, Yin Y, Hashem KM, MacGregor GA: **Gradual reduction of sugar in soft drinks without substitution as a strategy to reduce overweight, obesity, and type 2 diabetes: A modelling study**. *The Lancet Diabetes and Endocrinology* 2016, **4**(2):105-114.

23. Magnus A, Moodie ML, Ferguson M, Cobiac LJ, Liberato SC, Brimblecombe J: **The economic feasibility of price discounts to improve diet in Australian Aboriginal remote communities**. *Australian & New Zealand Journal of Public Health* 2016, **40**:S36-S41.

24. Manyema M, Veerman LJ, Chola L, Tugendhaft A, Sartorius B, Labadarios D, Hofman KJ: **The potential impact of a 20% tax on sugar-sweetened beverages on obesity in South African adults: A mathematical model**. *PLoS ONE* 2014, **9**(8).

25. Manyema M, Veerman JL, Chola L, Tugendhaft A, Labadarios D, Hofman K: **Decreasing the burden of type 2 diabetes in South Africa: The impact of taxing sugar-sweetened beverages**. *PLoS ONE* 2015, **10**(11).

26. Manyema M, Veerman LJ, Tugendhaft A, Labadarios D, Hofman KJ: **Modelling the potential impact of a sugar-sweetened beverage tax on stroke mortality, costs and health-adjusted life years in South Africa**. *BMC Public Health* 2016, **16**(1):1-10.

27. Mekonnen TA, Odden MC, Coxson PG, Guzman D, Lightwood J, Wang YC, Bibbins-Domingo K: **Health benefits of reducing sugar-sweetened beverage intake in high risk populations of California: Results from the Cardiovascular Disease (CVD) policy model**. *PLoS ONE* 2013, **8**(12).

28. Nomaguchi T, Cunich M, Zapata-Diomedi B, Veerman JL: **The impact on productivity of a hypothetical tax on sugar-sweetened beverages**. *Health Policy* 2017, **121**(6):715-725.

29. Pearson-Stuttard J, Bandosz P, Rehm CD, Penalvo J, Whitsel L, Gaziano T, Conrad Z, Wilde P, Micha R, Lloyd-Williams F *et al*: **Reducing us cardiovascular disease disparities through dietary policy**. *Circulation* 2017, **135**.

30. Penalvo JL, Cudhea F, Micha R, Rehm CD, Afshin A, Whitsel L, Wilde P, Gaziano T, Pearson-Stuttard J, O'Flaherty M *et al*: **The potential impact of food taxes and subsidies on cardiovascular disease and diabetes burden and disparities in the United States**. *BMC Medicine* 2017, **15**(1):208.

31. Rezende LF, Azeredo CM, Canella DS, Luiz Odo C, Levy RB, Eluf-Neto J: **Coronary heart disease mortality, cardiovascular disease mortality and all-cause mortality attributable to dietary intake over 20years in Brazil**. *International Journal of Cardiology* 2016, **217**:64-68.

32. Sacks G, Veerman JL, Moodie M, Swinburn B: **Traffic-light nutrition labelling and junk-food tax: A modelled comparison of cost-effectiveness for obesity prevention**. *International Journal of Obesity* 2011, **35**(7):1001-1009.

33. Sanchez-Romero LM, Penko J, Coxson PG, Fernandez A, Mason A, Moran AE, Avila-Burgos L, Odden M, Barquera S, Bibbins-Domingo K: **Projected Impact of Mexico's Sugar-Sweetened Beverage Tax Policy on Diabetes and Cardiovascular Disease: A Modeling Study**. *PLoS Medicine / Public Library of Science* 2016, **13**(11):e1002158.

34. Schwendicke F, Thomson WM, Broadbent JM, Stolpe M: **Effects of Taxing Sugar-Sweetened Beverages on Caries and Treatment Costs**. *Journal of Dental Research* 2016, **95**(12):1327-1332.

35. Singh GM, Micha R, Khatibzadeh S, Lim S, Ezzati M, Mozaffarian D: **Estimated Global, Regional, and National Disease Burdens Related to Sugar-Sweetened Beverage Consumption in 2010**. *Circulation* 2015, **132**(8):639-666.

36. Vecino-Ortiz AI, Arroyo-Ariza D: **A tax on sugar sweetened beverages in Colombia: Estimating the impact on overweight and obesity prevalence across socio economic levels**. *Social Science & Medicine* 2018, **209**:111-116.

37. Veerman JL, Sacks G, Antonopoulos N, Martin J: **The impact of a tax on sugar-sweetened beverages on health and health care costs: A modelling study**. *PLoS ONE* 2016, **11**(4).

38. Wang YC, Coxson P, Shen Y-M, Goldman L, Bibbins-Domingo K: **A Penny-Per-Ounce Tax On Sugar-Sweetened Beverages Would Cut Health And Cost Burdens Of Diabetes**. *Health Affairs* 2012, **31**(1):199-207.

39. Wilson KJ, Brown HS, Bastida E: **Cost-effectiveness of a community-based weight control intervention targeting a low-socioeconomic-status Mexican-origin population**. *Health promotion practice* 2015, **16**(1):101-108.

40. Wright DR, Kenney EL, Giles CM, Long MW, Ward ZJ, Resch SC, Moodie ML, Carter RC, Wang YC, Sacks G *et al*: **Modeling the Cost Effectiveness of Child Care Policy Changes in the U.S**. *American Journal of Preventive Medicine* 2015, **49**(1):135-147.
