# Supplementary material for: Sugar sweetened beverages attributable disease burden and the potential impact of policy interventions: a systematic review of epidemiological and decision models
Source: BMC Public Health. 2021 Jul 27;21:1460. doi: 10.1186/s12889-021-11046-7 (PMC8317409; doi:10.1186/s12889-021-11046-7)
Supplement: Supplementary file 3 — Additional file 3. Pathways by study [file 12889_2021_11046_MOESM3_ESM.pdf]

Sugar sweetened beverages attributable disease burden and the potential impact of policy interventions: a systematic review of epidemiological and decision models.
Additional file 3. Pathways by study.

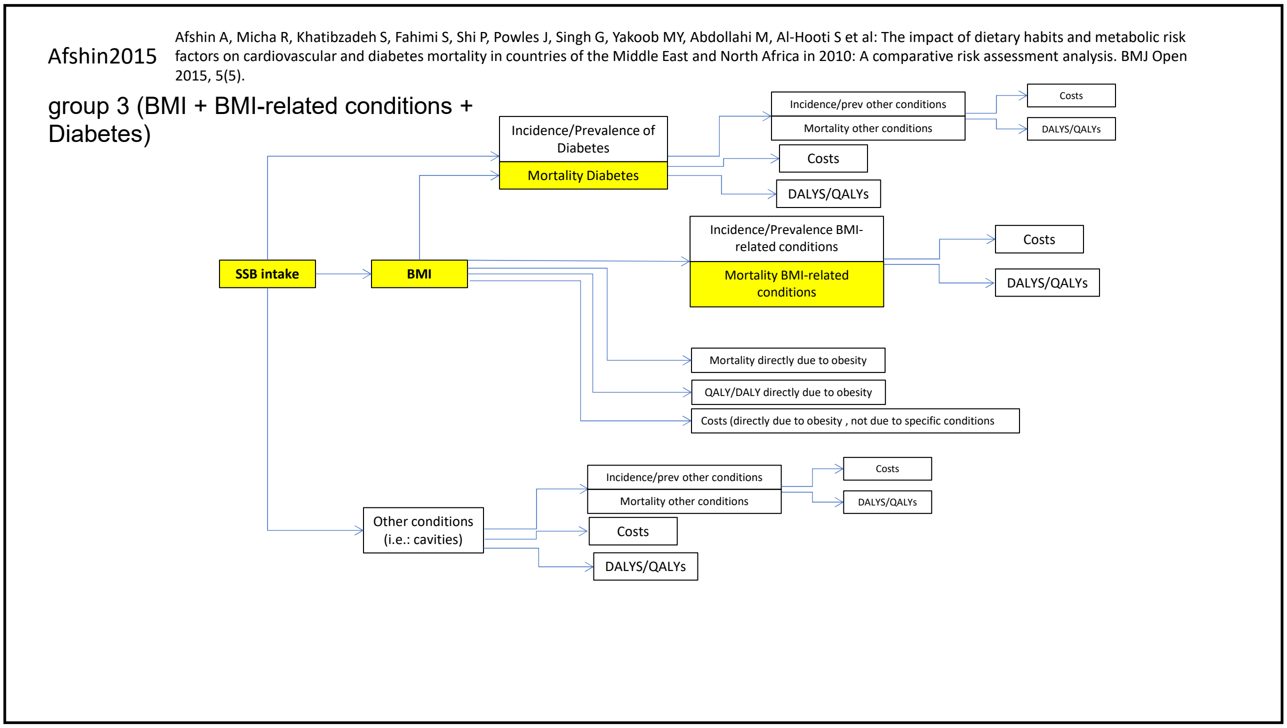

1

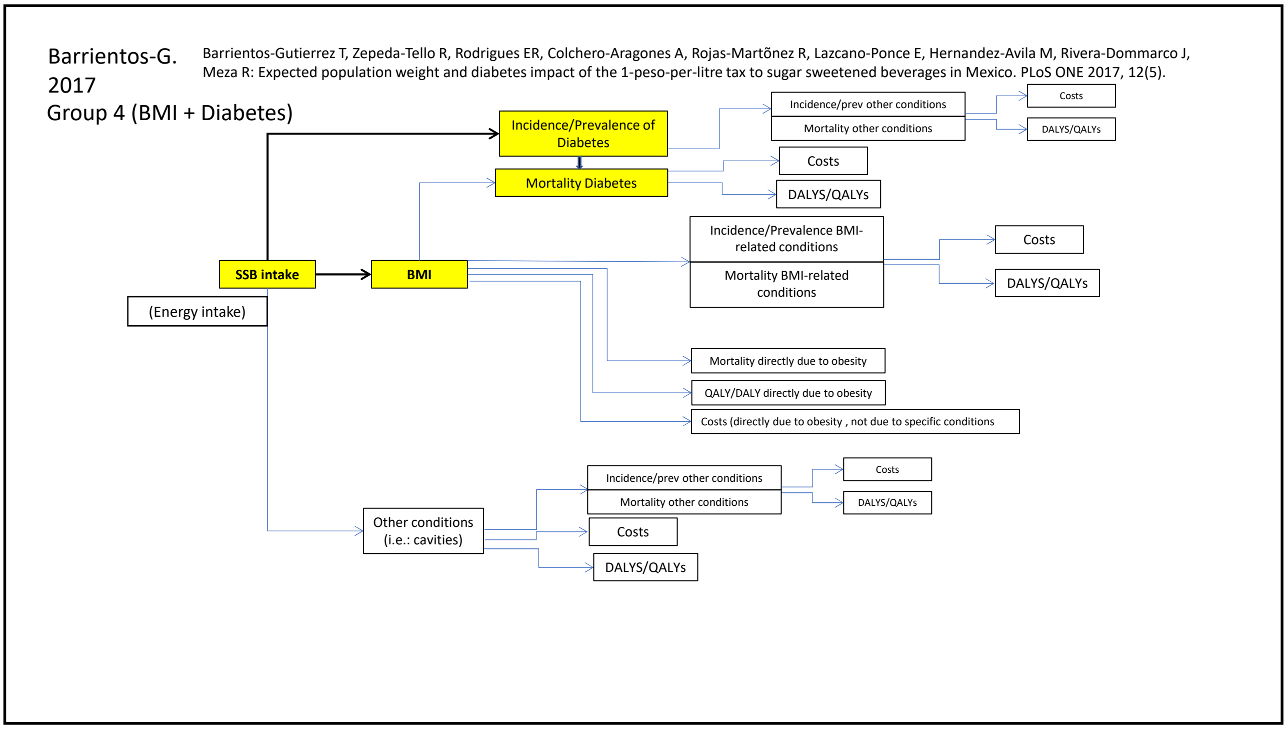

2

Basu 2013

Basu S, Seligman H, Bhattacharya J: **Nutritional policy changes in the supplemental nutrition assistance program: a microsimulation and cost-effectiveness analysis**. *Medical Decision Making* 2013, **33**(7):937-948.

group 3 (BMI + BMI-related conditions + Diabetes)

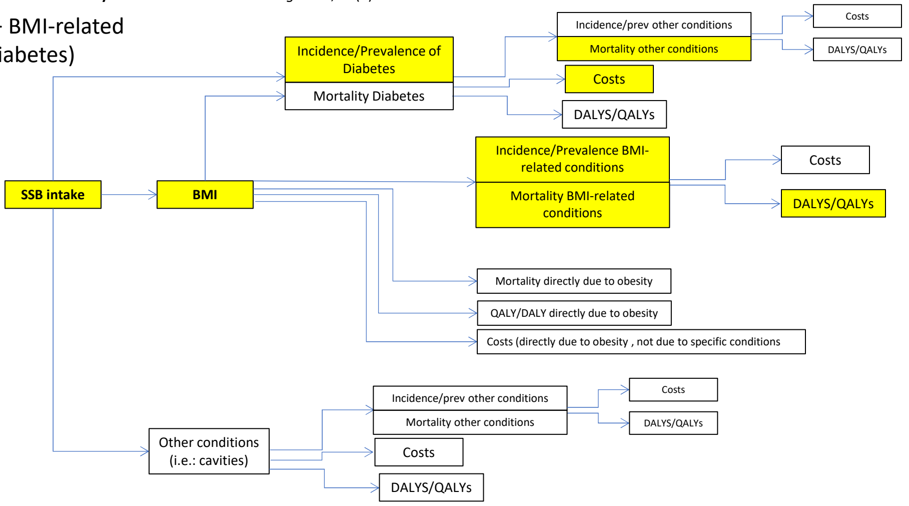

3

Basu 2014 a

Basu S, Vellakkal S, Agrawal S, Stuckler D, Popkin B, Ebrahim S: **Averting obesity and type 2 diabetes in India through sugar-sweetened beverage taxation: an economic-epidemiologic modeling study**. *PLoS Medicine* 2014, **11**(1):e1001582-e1001582.

Group 4 (BMI + Diabetes)

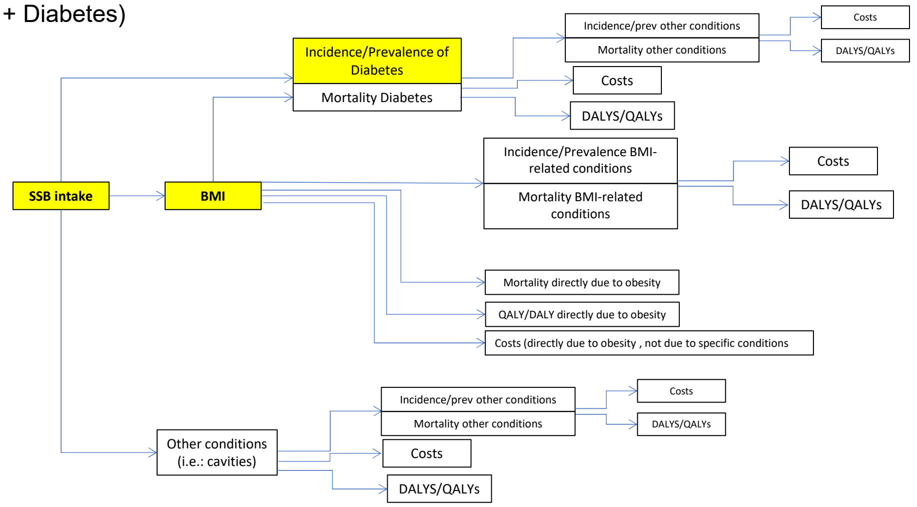

4

Basu 2014 b

Basu S, Lewis K: Reducing Added Sugars in the Food Supply Through a Cap-and-Trade Approach. American Journal of Public Health 2014, 104(12):2432-2438.

Group 4 (BMI + Diabetes)

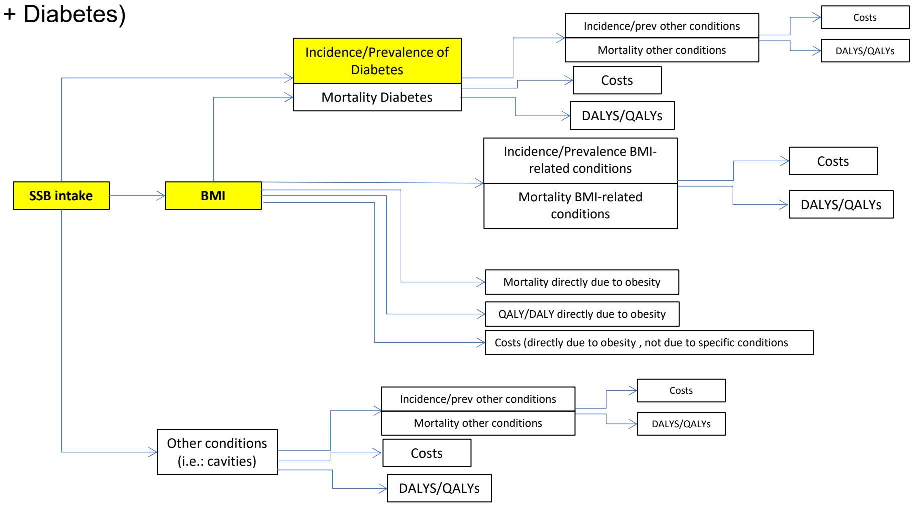

5

Breeze2017

Breeze PR, Thomas C, Squires H, Brennan A, Greaves C, Diggle P, Brunner E, Tabak A, Preston L, Chilcott J: Cost-effectiveness of population-based, community, workplace and individual policies for diabetes prevention in the UK. Diabetic Medicine 2017, 34(8):1136-1144.

group 3 (BMI + BMI-related conditions + Diabetes)

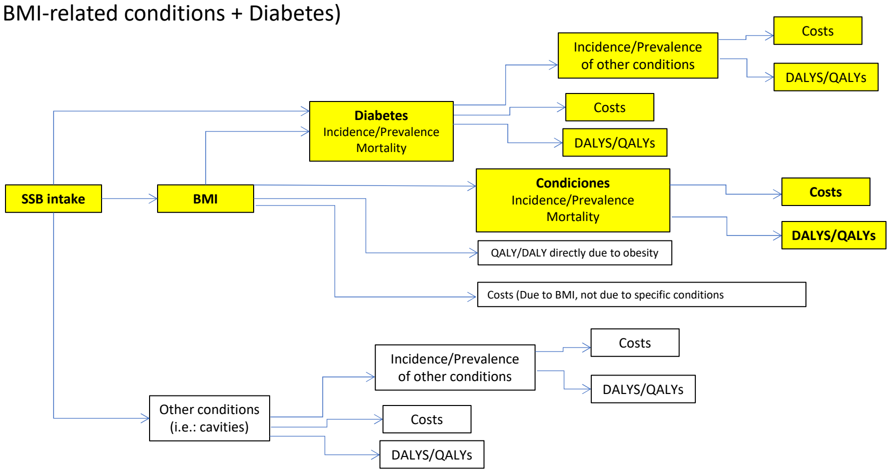

6

Briggs 2013a

Briggs AD, Mytton OT, Kehlbacher A, Tiffin R, Rayner M, Scarborough P: Overall and income specific effect on prevalence of overweight and obesity of 20% sugar sweetened drink tax in UK: econometric and comparative risk assessment modelling study. BMJ 2013, 347:f6189.

Group 1 (Only BMI)

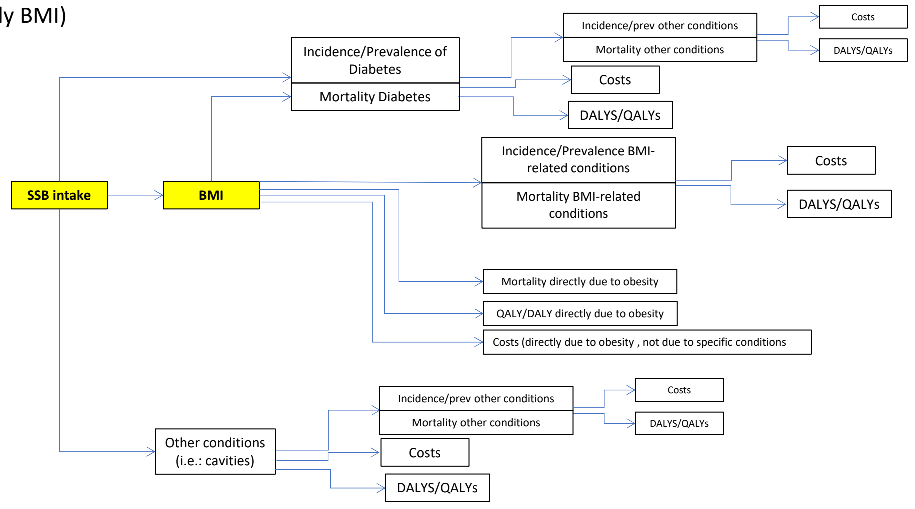

7

Briggs 2013b

Briggs AD, Mytton OT, Madden D, O'Shea D, Rayner M, Scarborough P: The potential impact on obesity of a 10% tax on sugar-sweetened beverages in Ireland, an effect assessment modelling study. BMC public health 2013, 13:860.

Group 1 (Only BMI)

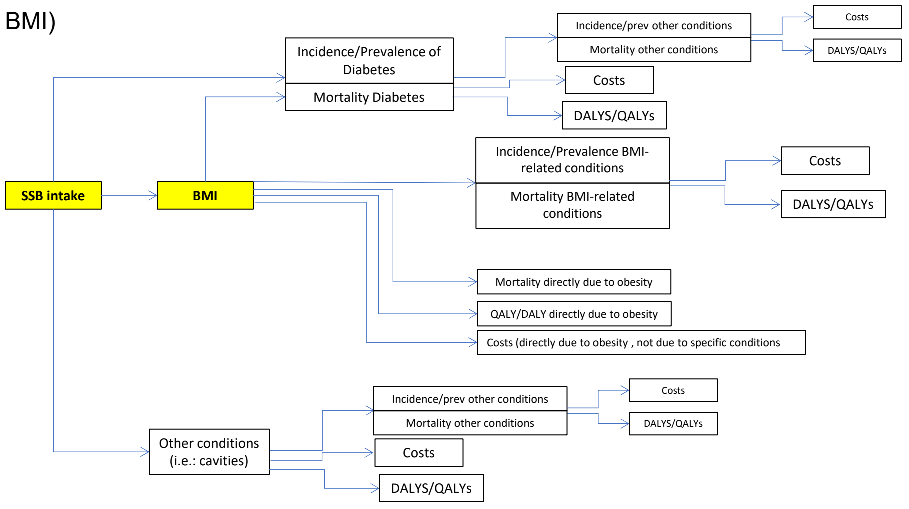

8

Briggs 2017

Briggs ADM, Mytton OT, Kehlbacher A, Tiffin R, Elhussein A, Rayner M, Jebb SA, Blakely T, Scarborough P: **Health impact assessment of the UK soft drinks industry levy: a comparative risk assessment modelling study**. *The Lancet Public Health* 2017, **2**(1):e15-e22.

group 3 (BMI + BMI-related conditions + Diabetes)

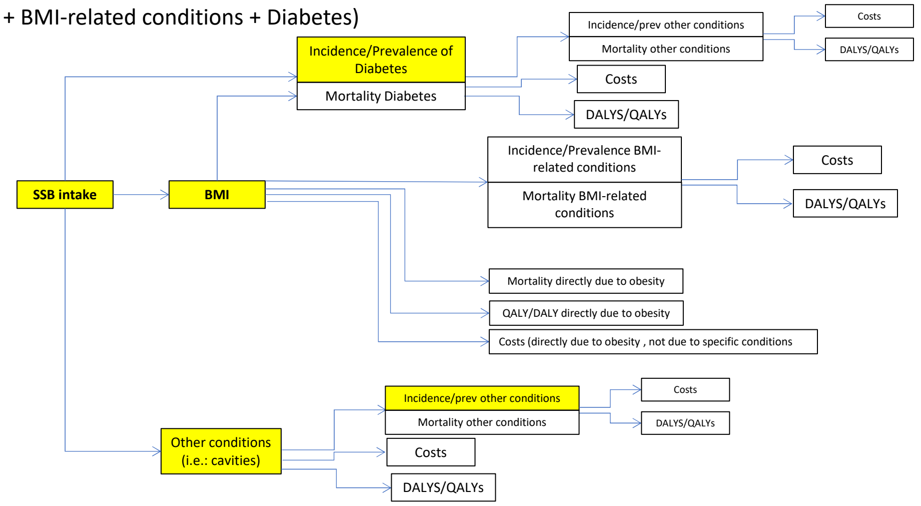

9

Brown 2018

Brown V, Ananthapavan J, Veerman L, Sacks G, Lal A, Peeters A, Backholer K, Moodie M: The Potential Cost-Effectiveness and Equity Impacts of Restricting Television Advertising of Unhealthy Food and Beverages to Australian Children. Nutrients 2018, 10(5):N.PAG-N.PAG.

group 3 (BMI + BMI-related conditions + Diabetes)

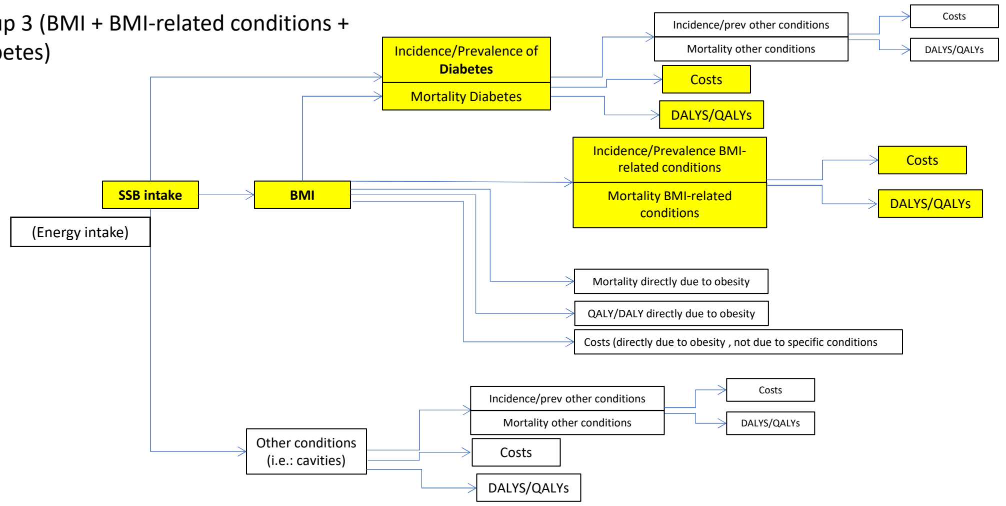

10

Cobiac 2017 Cobiac LJ, Tam K, Veerman L, Blakely T: Taxes and Subsidies for Improving Diet and Population Health in Australia: A Cost-Effectiveness Modelling Study. PLoS Medicine 2017, 14(2).

group 3 (BMI + BMI-related conditions + Diabetes)

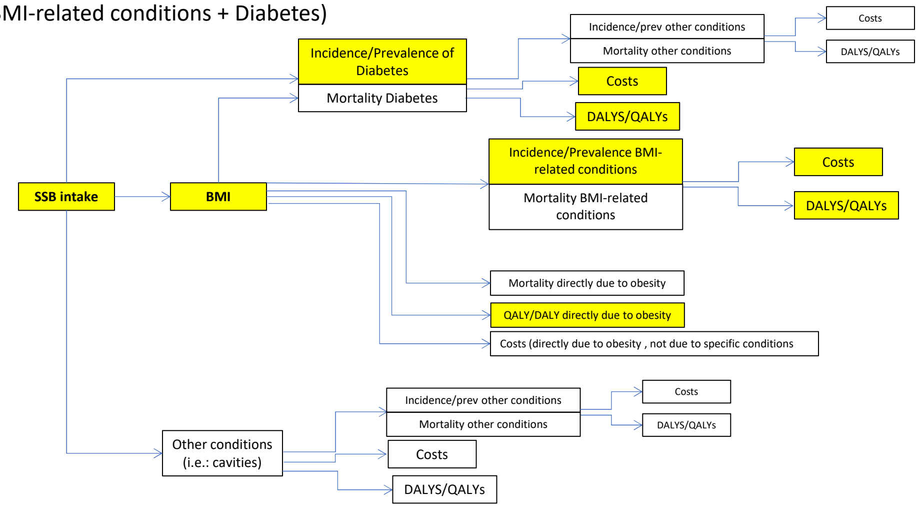

11

Collins 2015 Collins B, Capewell S, O'Flaherty M, Timpson H, Razzaq A, Cheater S, Ireland R, Bromley H: Modelling the Health Impact of an English Sugary Drinks Duty at National and Local Levels. PLoS ONE [Electronic Resource] 2015, 10(6):e0130770.

Group 2 (BMI + BMI-related conditions /consequences)

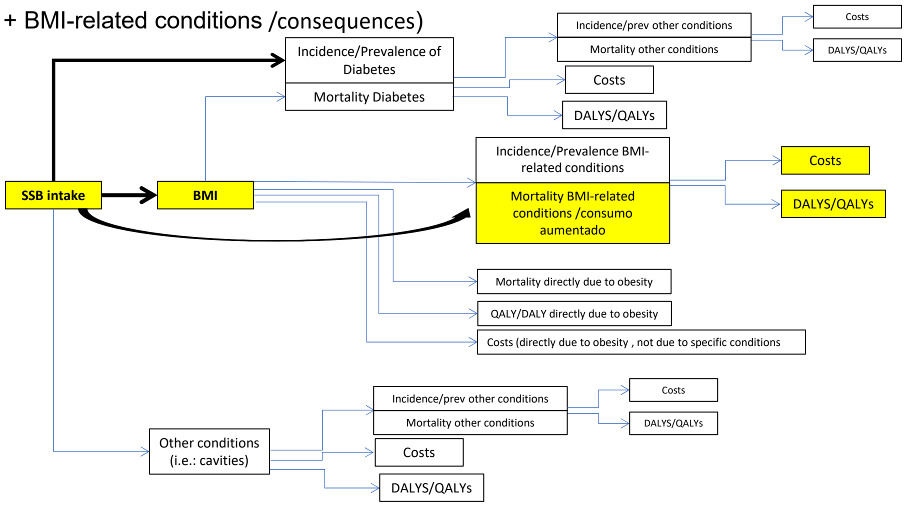

12

Crino 2017* Crino M, Mantilla Herrera AM, Ananthapavan J, Wu JHY, Neal B, Yong Yi L, Miaobing Z, Lal A, Sacks G: Modelled Cost-Effectiveness of a Package Size Cap and a Kilojoule Reduction Intervention to Reduce Energy Intake from Sugar-Sweetened Beverages in Australia. Nutrients 2017, 9(9):1-17.

group 3 (BMI + BMI-related conditions + Diabetes)

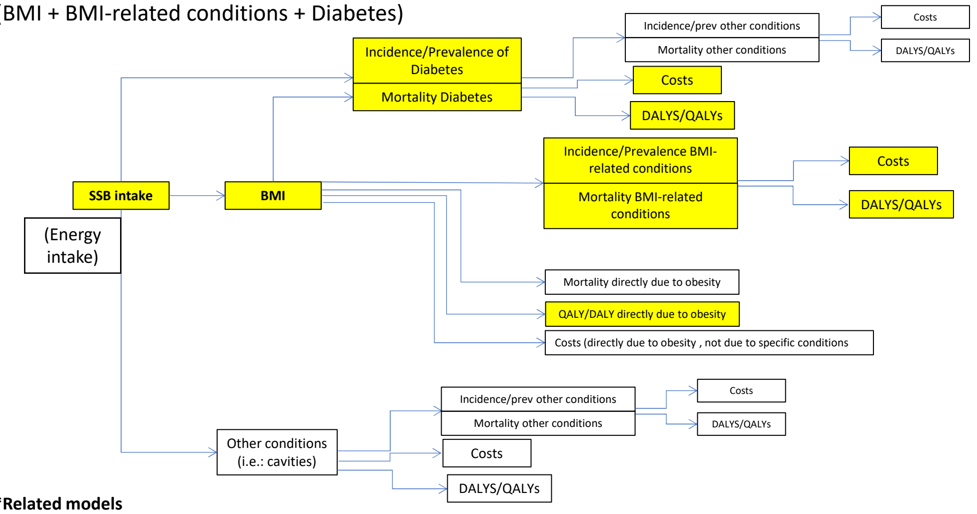

***Related models**

Carter R, Moodie M, Markwick A, Magnus A, Vos T, Swinburn B, et al. Assessing cost-effectiveness in obesity (ACE-obesity): an overview of the ACE approach, economic methods and cost results. BMC Public Health. 2009; 9:419.

Vos T, Carter R, Doran C, Anderson I, Lopez A, Wilson A. Assessing cost-effectiveness in the prevention of non-communicable disease (ACE-Prevention) project 2005±09: economic evaluation protocol (as per September 2007). Brisbane: University of Queensland; 2007.

13

Gortmaker 2015a **a**. Gortmaker SL, Long MW, Resch SC, Ward ZJ, Cradock AL, Barrett JL, Wright DR, Sonneville KR, Giles CM, Carter RC et al: Cost Effectiveness of Childhood Obesity Interventions: Evidence and Methods for CHOICES. American Journal of Preventive Medicine 2015, 49(1):102-111.

Group 2 (BMI + BMI-related conditions /consequences)

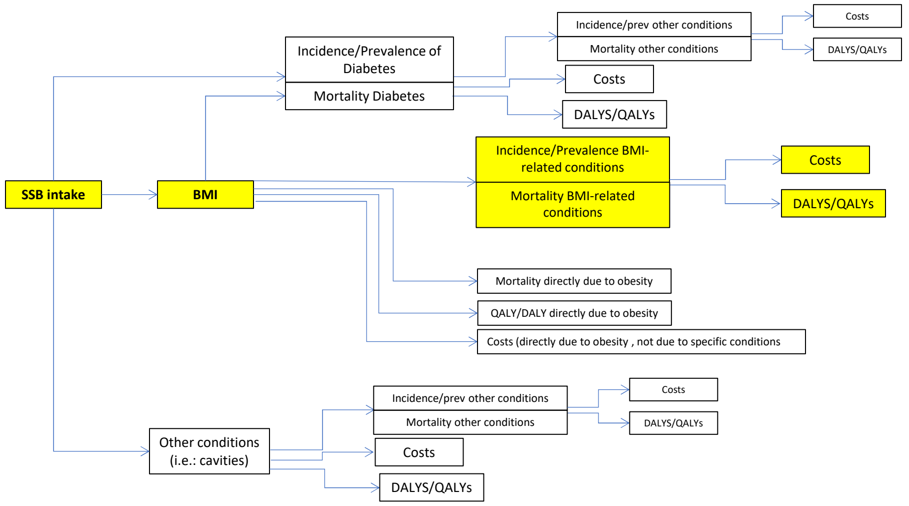

14

Gortmaker 2015b

b. Gortmaker SL, Wang YC, Long MW, Giles CM, Ward ZJ, Barrett JL, Kenney EL, Sonneville KR, Sadaf Afzal A, Resch SC et al: Three Interventions That Reduce Childhood Obesity Are Projected To Save More Than They Cost To Implement. Health Affairs 2015, 34(11):1932-1939.

Group 2 (BMI + BMI-related conditions /consequences)

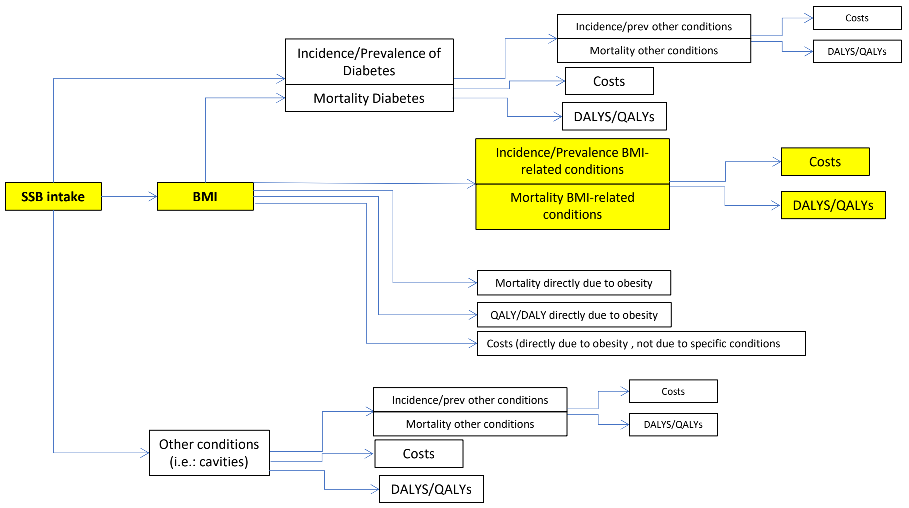

15

Kristensen2014

Kristensen AH, Flottemesch TJ, Maciosek MV, Jenson J, Barclay G, Ashe M, Sanchez EJ, Story M, Teutsch SM, Brownson RC: **Reducing childhood obesity through U.S. federal policy: a microsimulation analysis**. *American Journal of Preventive Medicine* 2014, **47**(5):604-612.

Group 1 (Only BMI)

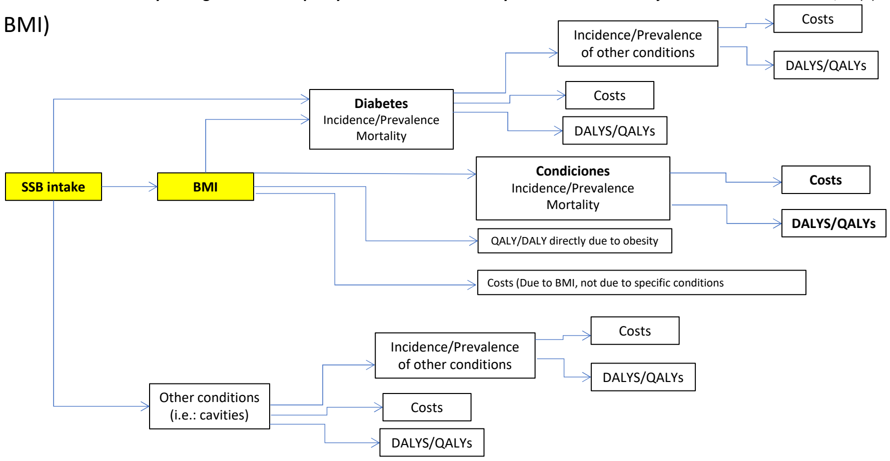

16

Lal 2017*

Kristensen AH, Flottemesch TJ, Maciosek MV, Jenson J, Barclay G, Ashe M, Sanchez EJ, Story M, Teutsch SM, Brownson RC: Reducing childhood obesity through U.S. federal policy: a microsimulation analysis. American Journal of Preventive Medicine 2014, 47(5):604-612.

group 3 (BMI + BMI-related conditions + Diabetes)

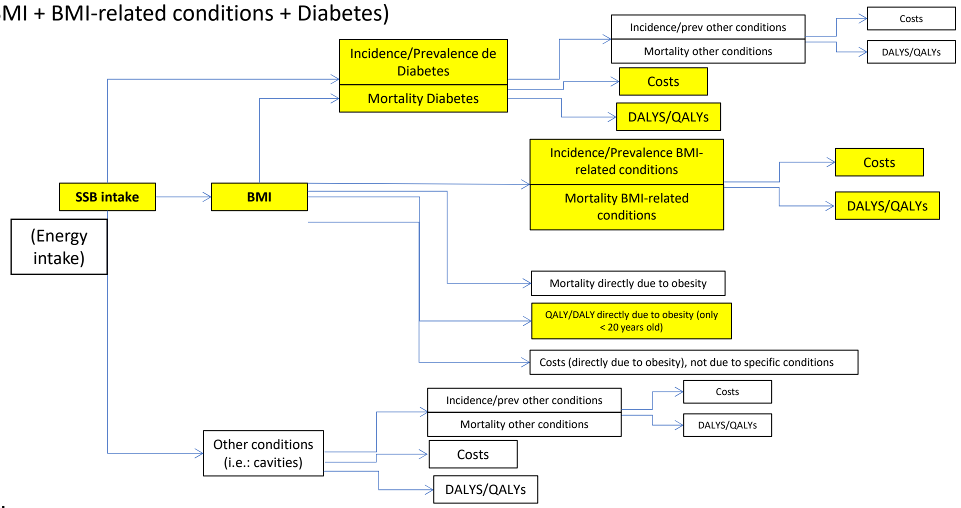

***Related papers**

Carter R, Moodie M, Markwick A, Magnus A, Vos T, Swinburn B, et al. Assessing cost-effectiveness in obesity (ACE-obesity): an overview of the ACE approach, economic methods and cost results. BMC Public Health. 2009; 9:419. https://doi.org/10.1186/1471-2458-9-419 PMID: 19922625

Vos T, Carter R, Doran C, Anderson I, Lopez A, Wilson A. Assessing cost-effectiveness in the prevention of non-communicable disease (ACE-Prevention) project 2005±09: economic evaluation protocol (as per September 2007). Brisbane: University of Queensland; 2007.

17

Lee 2018

Lee BY, Ferguson MC, Hertenstein DL, Adam A, Zenkov E, Wang PI, Wong MS, Gittelsohn J, Mui Y, Brown ST: Simulating the Impact of Sugar-Sweetened Beverage Warning Labels in Three Cities. American Journal of Preventive Medicine 2018, 54(2):197-204.

Group 1 (Only BMI)

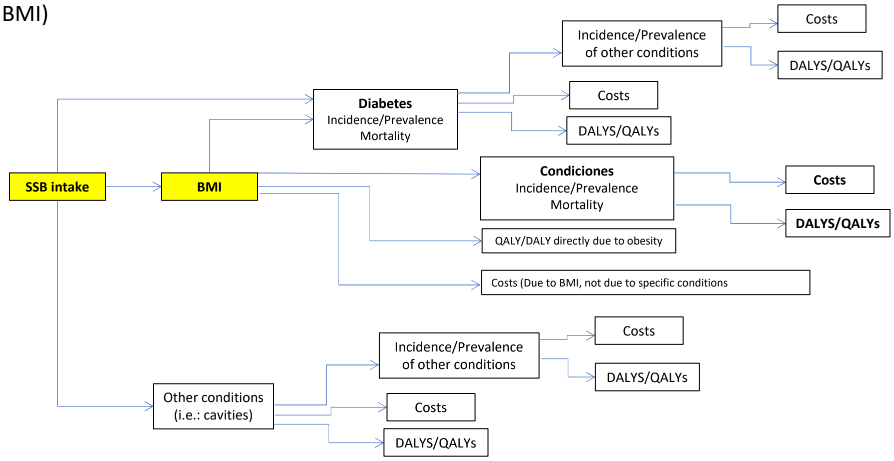

18

Lieffers 2018

Lieffers JRL, Ekwaru JP, Ohinmaa A, Veugelers PJ: The economic burden of not meeting food recommendations in Canada: The cost of doing nothing. PLoS ONE 2018, 13(4).

Group 6 (Other → any other)

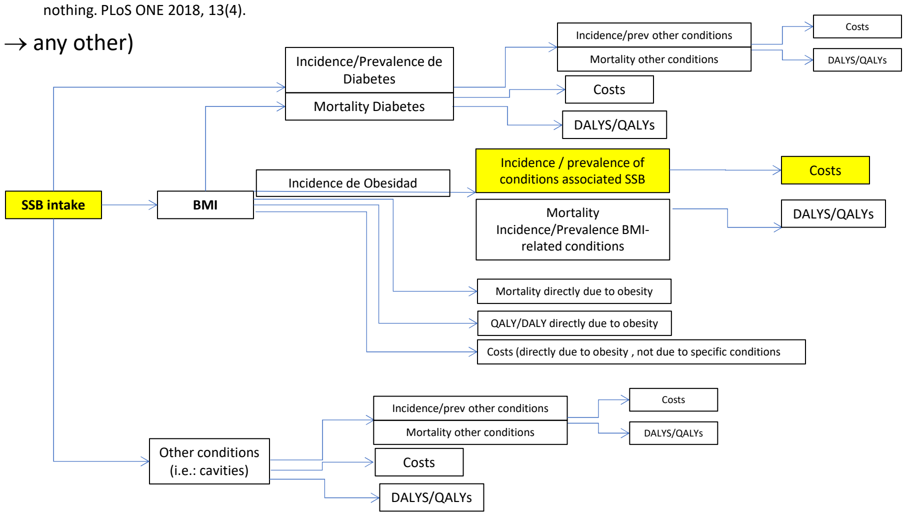

19

Lin 2011

Lin BH, Smith TA, Lee JY, Hall KD: Measuring weight outcomes for obesity intervention strategies: The case of a sugar-sweetened beverage tax. Economics and Human Biology 2011, 9(4):329-341.

Group 2 (BMI + BMI-related conditions /consequences)

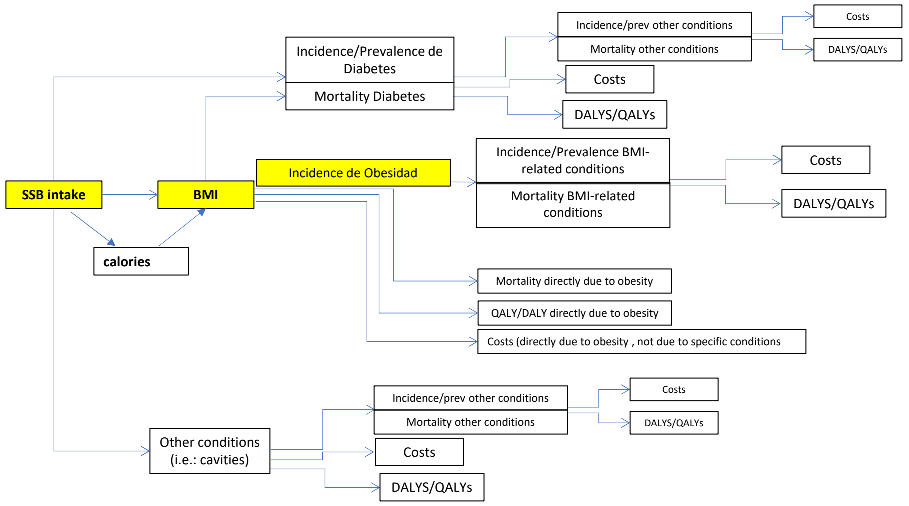

20

Long 2015 Long MW, Gortmaker SL, Ward ZJ, Resch SC, Moodie ML, Sacks G, Swinburn BA, Carter RC, Claire Wang Y: Cost Effectiveness of a Sugar-Sweetened Beverage Excise Tax in the U.S. American Journal of Preventive Medicine 2015, 49(1):112-123.

Group 2 (BMI + BMI-related conditions /consequences)

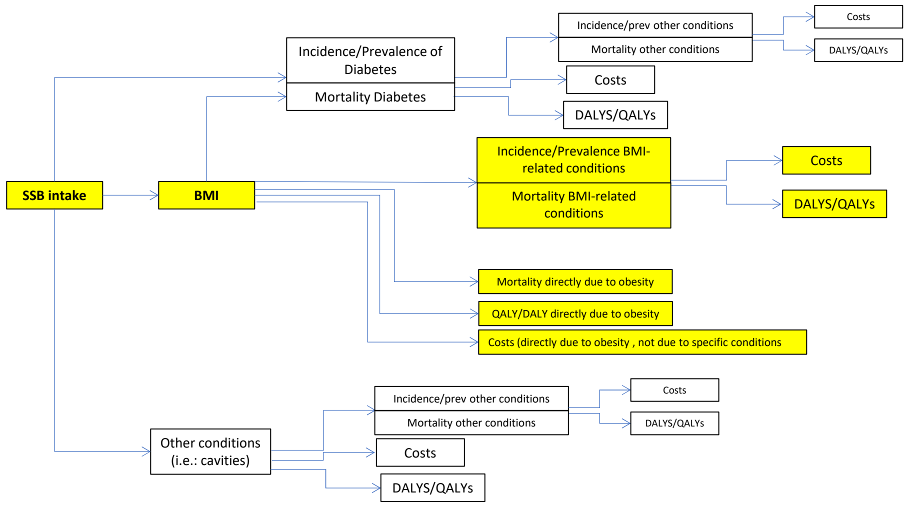

21

Ma 2016 Ma Y, He FJ, Yin Y, Hashem KM, MacGregor GA: Gradual reduction of sugar in soft drinks without substitution as a strategy to reduce overweight, obesity, and type 2 diabetes: A modelling study. The Lancet Diabetes and Endocrinology 2016, 4(2):105-114.

Group 4 (BMI + Diabetes)

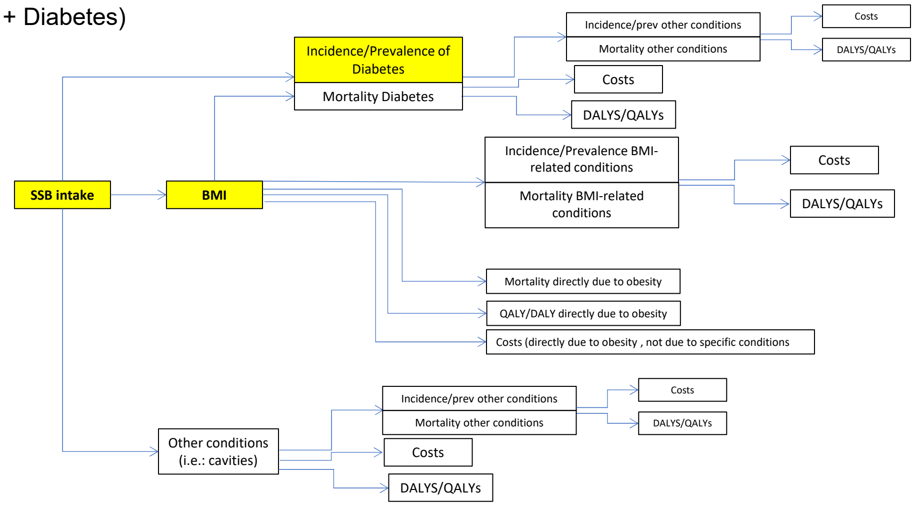

22

Magnus 2016* Magnus A, Moodie ML, Ferguson M, Cobiac LJ, Liberato SC, Brimblecombe J: The economic feasibility of price discounts to improve diet in Australian Aboriginal remote communities. Australian & New Zealand Journal of Public Health 2016, 40:S36-S41

group 3 (BMI + BMI-related conditions + Diabetes)

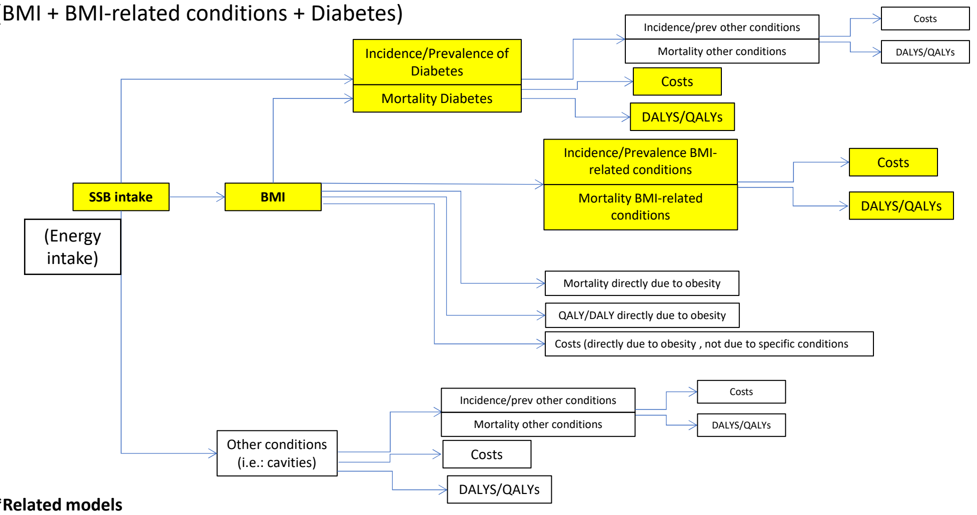

***Related models**

Cobiac L, Vos T, Veerman L. Cost-effectiveness of Weight Watchers and the Lighten Up to a Healthy Lifestyle program. Aust N Z J Public Health. 2010;34(3):240-7

23

Manyema 2014 Manyema M, Veerman LJ, Chola L, Tugendhaft A, Sartorius B, Labadarios D, Hofman KJ: The potential impact of a 20% tax on sugar-sweetened beverages on obesity in South African adults: A mathematical model. PLoS ONE 2014, 9(8).

Group 1 (Only BMI)

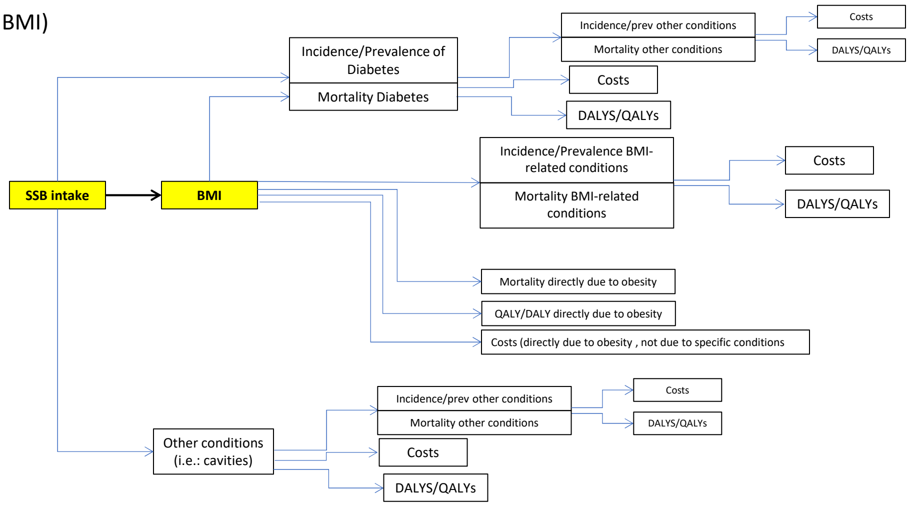

24

Manyema 2015

Manyema M, Veerman JL, Chola L, Tugendhaft A, Labadarios D, Hofman K: Decreasing the burden of type 2 diabetes in South Africa: The impact of taxing sugar-sweetened beverages. PLoS ONE 2015, 10(11).

Group 2 (BMI + BMI-related conditions/consequences)

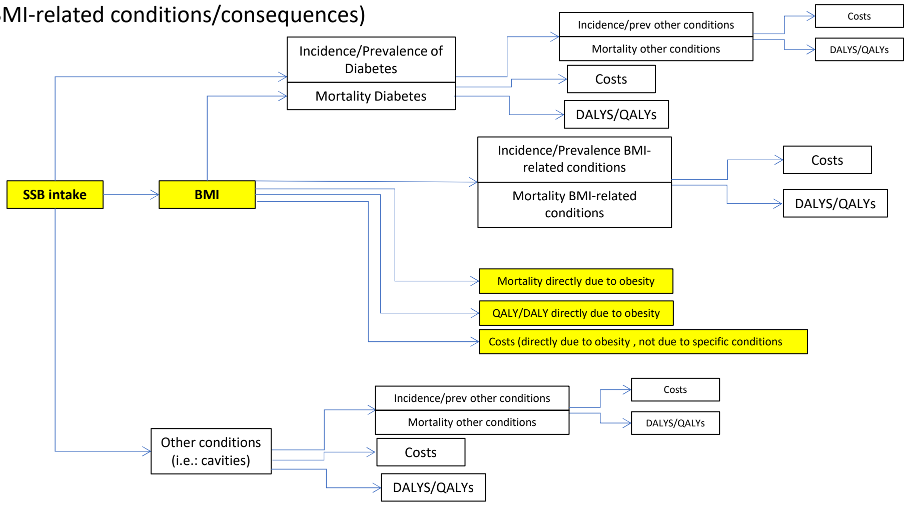

25

Manyema 2016

Manyema M, Veerman LJ, Tugendhaft A, Labadarios D, Hofman KJ: Modelling the potential impact of a sugar-sweetened beverage tax on stroke mortality, costs and health-adjusted life years in South Africa. BMC Public Health 2016, 16(1):1-10.

Group 2 (BMI + BMI-related conditions /consequences)

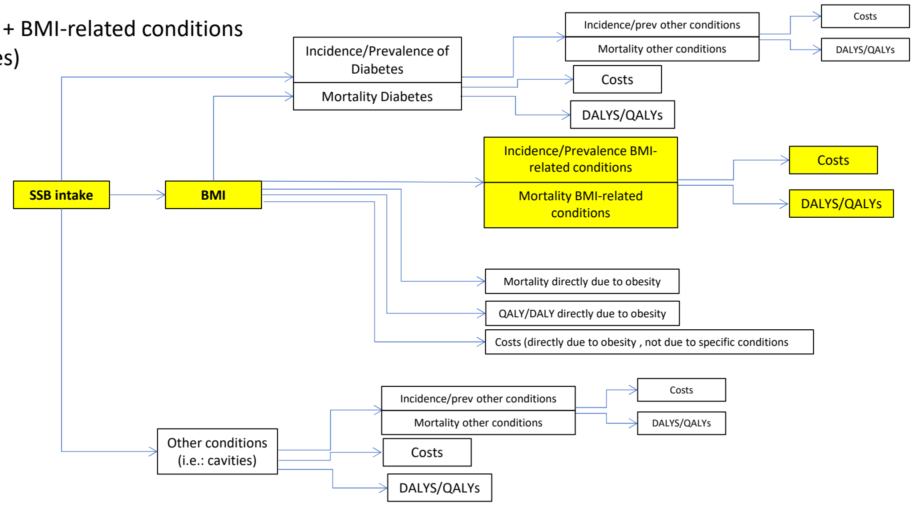

26

Mekonnen 2013

Mekonnen TA, Odden MC, Coxson PG, Guzman D, Lightwood J, Wang YC, Bibbins-Domingo K: **Health benefits of reducing sugar-sweetened beverage intake in high risk populations of California: Results from the Cardiovascular Disease (CVD) policy model**. *PLoS ONE* 2013, **8**(12).

group 3 (BMI + BMI-related conditions + Diabetes)

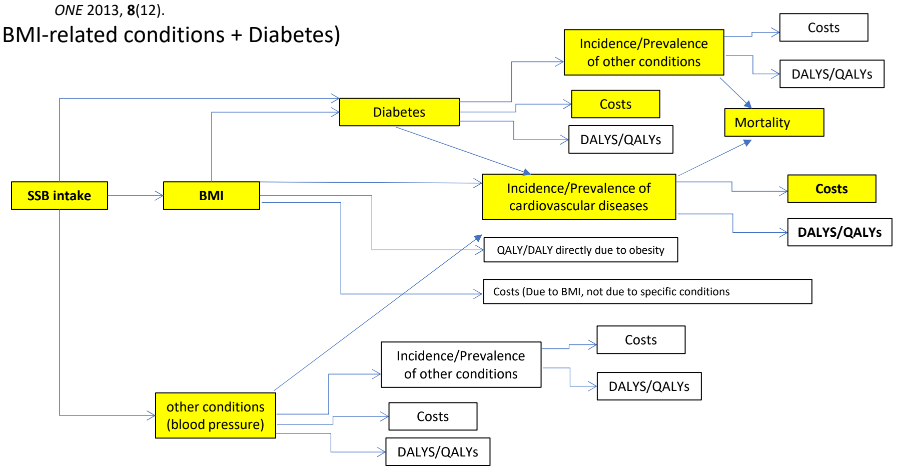

27

Nomaguchi 2017

Nomaguchi T, Cunich M, Zapata-Diomedi B, Veerman JL: **The impact on productivity of a hypothetical tax on sugar-sweetened beverages**. *Health Policy* 2017, **121**(6):715-725.

Group 2 (BMI + BMI-related conditions /consequences)

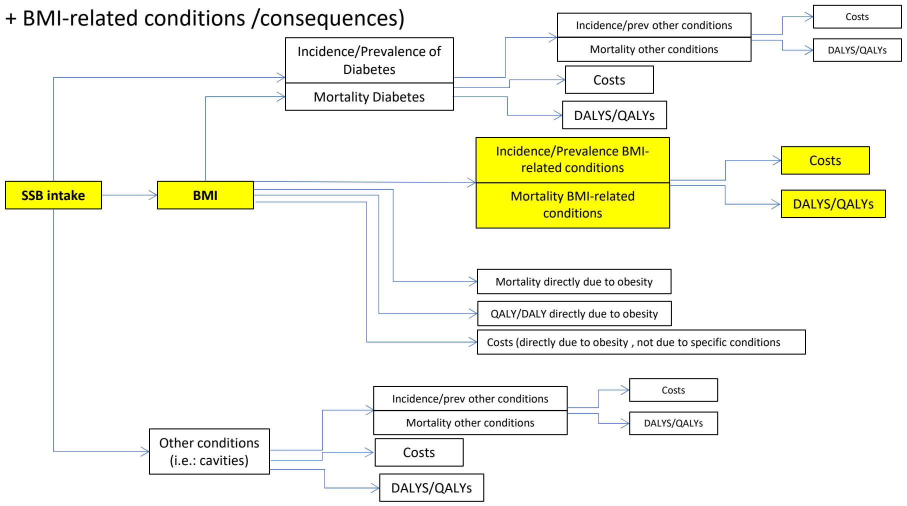

28

Pearson-Stuttard 2017

Pearson-Stuttard J, Bandosz P, Rehm CD, Penalvo J, Whitsel L, Gaziano T, Conrad Z, Wilde P, Micha R, Lloyd-Williams F et al: Reducing us cardiovascular disease disparities through dietary policy. Circulation 2017, 135.

Group 2 (BMI + BMI-related conditions /consequences)

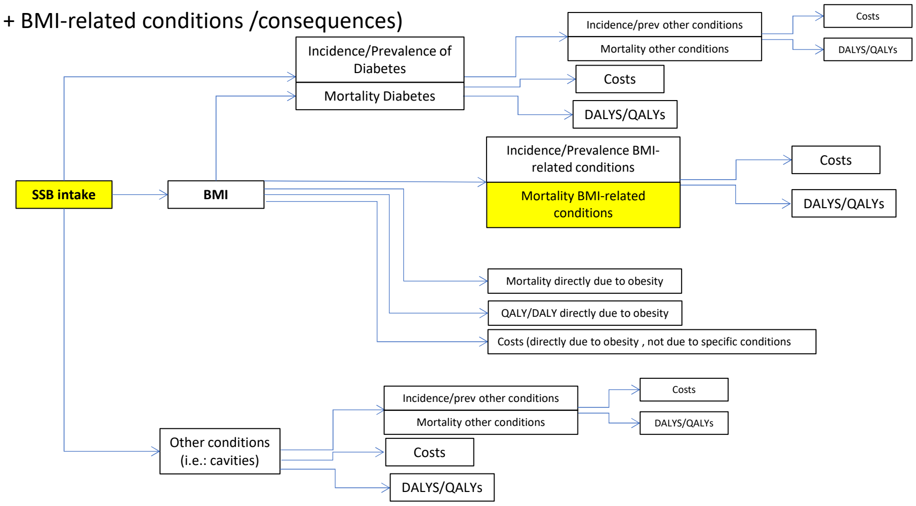

29

Penalvo 2017

Penalvo JL, Cudhea F, Micha R, Rehm CD, Afshin A, Whitsel L, Wilde P, Gaziano T, Pearson-Stuttard J, O'Flaherty M et al: The potential impact of food taxes and subsidies on cardiovascular disease and diabetes burden and disparities in the United States. BMC Medicine, 15(1):208

group 3 (BMI + BMI-related conditions + Diabetes)

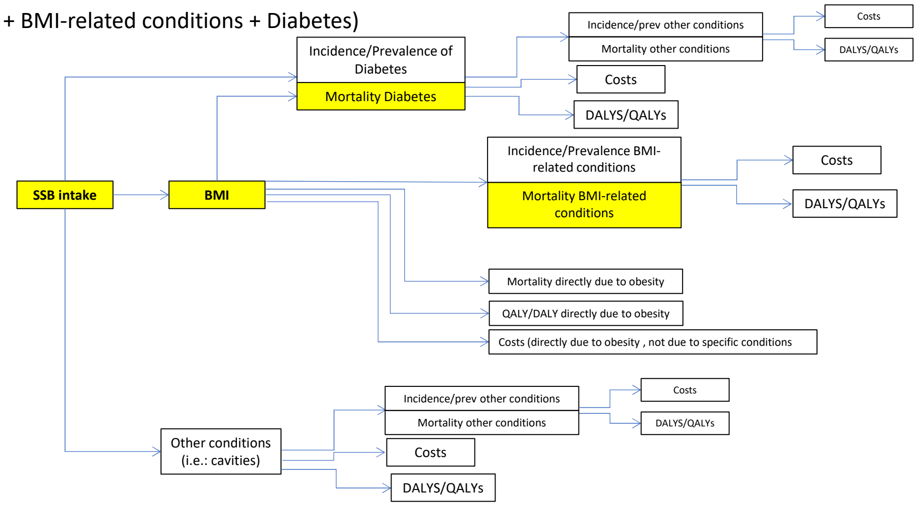

30

Rezende 2016

Rezende LF, Azeredo CM, Canella DS, Luiz Odo C, Levy RB, Eluf-Neto J: Coronary heart disease mortality, cardiovascular disease mortality and all-cause mortality attributable to dietary intake over 20years in Brazil. International Journal of Cardiology, 217:64-68.

Group 2 (BMI + BMI-related conditions /consequences)

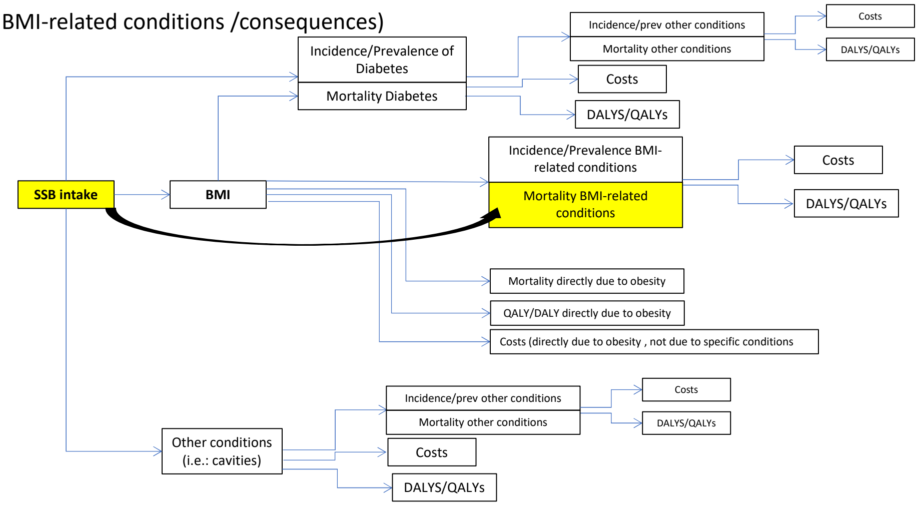

31

Sacks 2011

Sacks G, Veerman JL, Moodie M, Swinburn B: Traffic-light nutrition labelling and junk-food tax: A modelled comparison of cost-effectiveness for obesity prevention. International Journal of Obesity 2011, 35(7):1001-1009

Group 2 (BMI + BMI-related conditions /consequences)

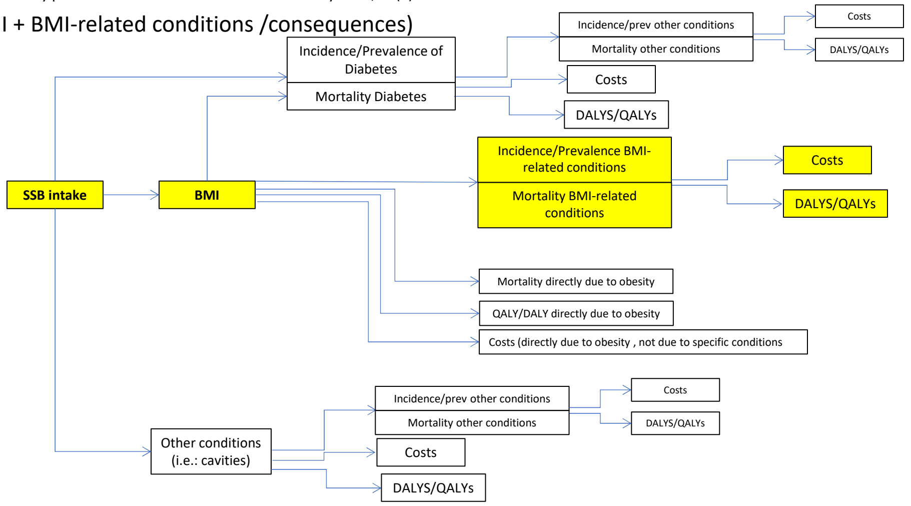

32

Sanchez Romero 2016

Sanchez-Romero LM, Penko J, Coxson PG, Fernandez A, Mason A, Moran AE, Avila-Burgos L, Odden M, Barquera S, Bibbins-Domingo K: Projected Impact of Mexico's Sugar-Sweetened Beverage Tax Policy on Diabetes and Cardiovascular Disease: A Modeling Study. PLoS Medicine / Public Library of Science, 13(11):e1002158.

group 3 (BMI + BMI-related conditions + Diabetes)

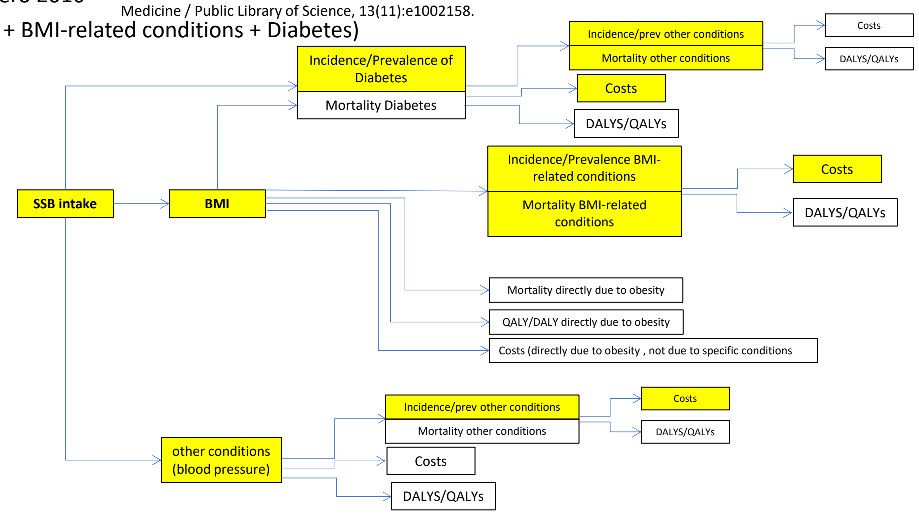

33

Schwendicke 2016

Schwendicke F, Thomson WM, Broadbent JM, Stolpe M: Effects of Taxing Sugar-Sweetened Beverages on Caries and Treatment Costs. Journal of Dental Research 2016, 95(12):1327-1332.

group 5 (Other, such as cavities, → costs)

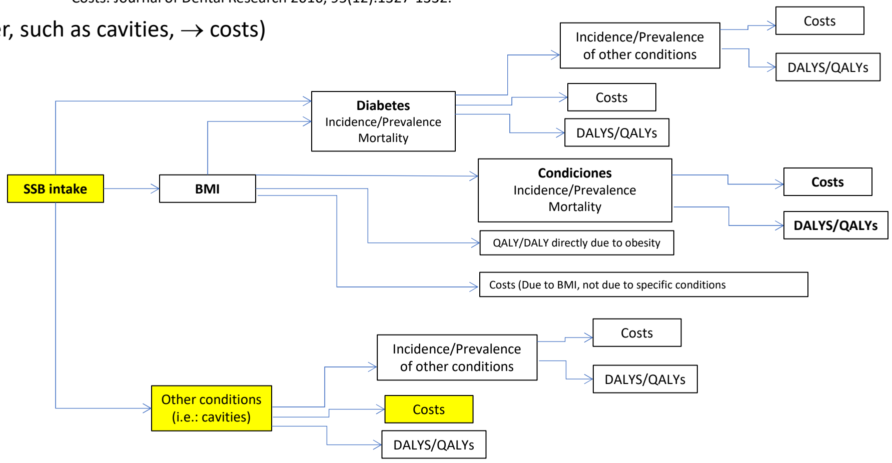

34

Singh 2015 Singh GM, Micha R, Khatibzadeh S, Lim S, Ezzati M, Mozaffarian D: Estimated Global, Regional, and National Disease Burdens Related to Sugar-Sweetened Beverage Consumption in 2010. Circulation 2015, 132(8):639-666.

Group 2 (BMI + BMI-related conditions /consequences)

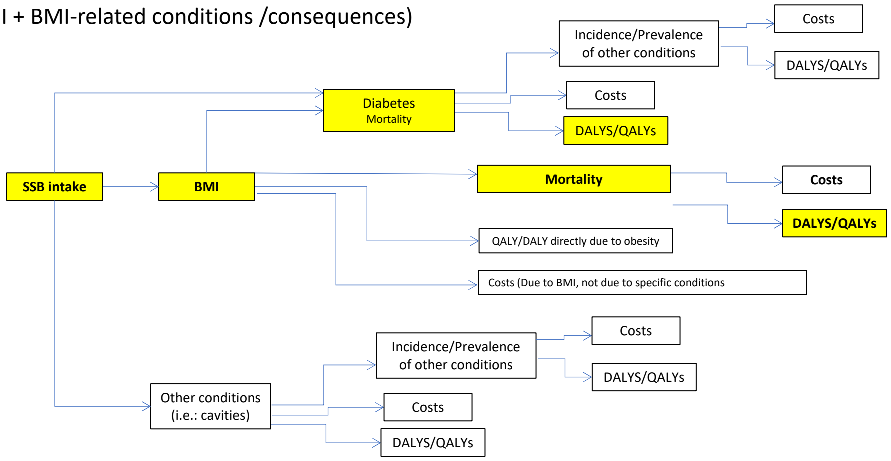

35

Vecino-Ortiz 2018 Vecino-Ortiz AI, Arroyo-Ariza D: A tax on sugar sweetened beverages in Colombia: Estimating the impact on overweight and obesity prevalence across socio economic levels. Social Science & Medicine 2018, 209:111-116.

Group 1 (Only BMI)

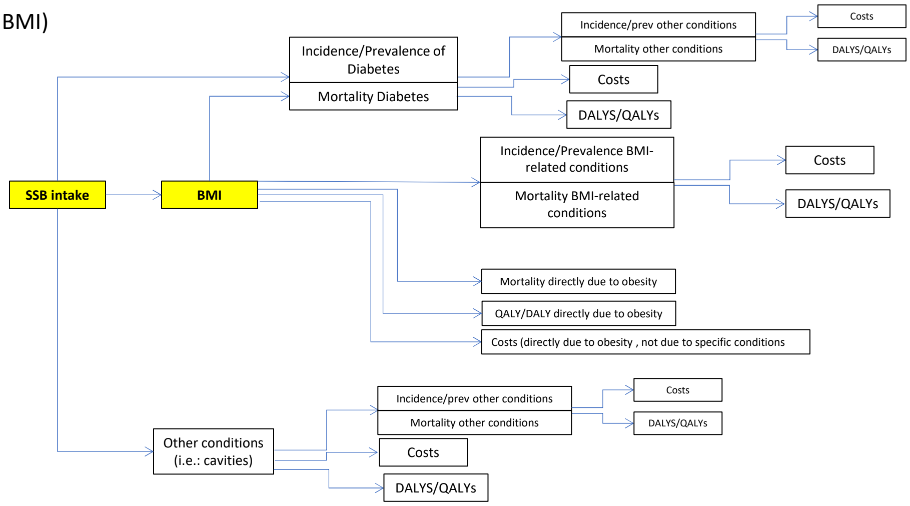

36

Veerman 2016

Veerman JL, Sacks G, Antonopoulos N, Martin J: The impact of a tax on sugar-sweetened beverages on health and health care costs: A modelling study. PLoS ONE 2016, 11(4).

group 3 (BMI + BMI-related conditions + Diabetes)

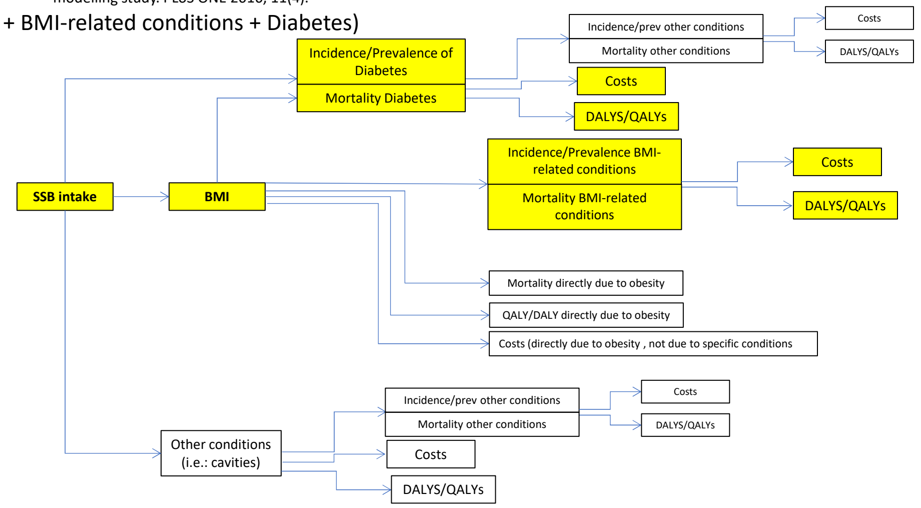

37

Wang 2012

Wang YC, Coxson P, Shen Y-M, Goldman L, Bibbins-Domingo K: A Penny-Per-Ounce Tax On Sugar-Sweetened Beverages Would Cut Health And Cost Burdens Of Diabetes. Health Affairs 2012, 31(1):199-207.

group 3 (BMI + BMI-related conditions + Diabetes)

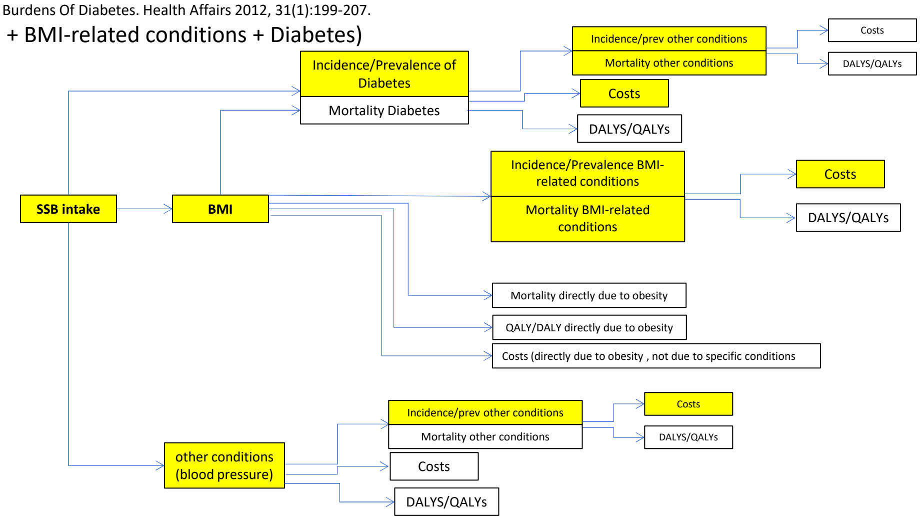

38

Wilson 2015
(Archimides model)
Group 1 (Only BMI)

Wilson KJ, Brown HS, Bastida E: Cost-effectiveness of a community-based weight control intervention targeting a low-socioeconomic-status Mexican-origin population. Health promotion practice 2015, 16(1):101-108.

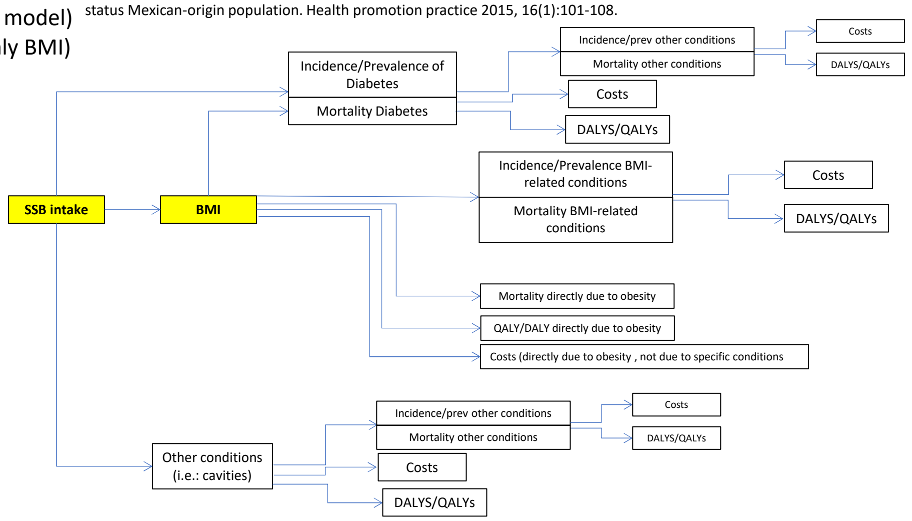

39

Wright 2015

Wright DR, Kenney EL, Giles CM, Long MW, Ward ZJ, Resch SC, Moodie ML, Carter RC, Wang YC, Sacks G et al: Modeling the Cost Effectiveness of Child Care Policy Changes in the U.S. American Journal of Preventive Medicine 2015, 49(1):135-147.

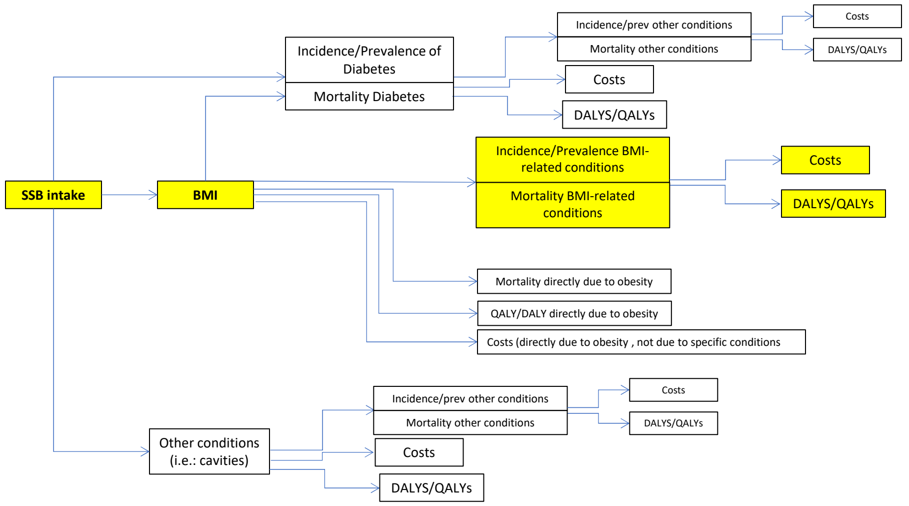

40
